# Electro‐Chemo‐Mechanical Coupling in Composite Cathodes of Sulfide‐Based All‐Solid‐State Batteries: Pathways, Degradation, and Design Rules

**DOI:** 10.1002/advs.202524187

**Published:** 2026-02-13

**Authors:** Gawon Song, Seonghyun Lee, Minseon Lee, Junsung Park, Kyu Tae Lee

**Affiliations:** ^1^ School of Chemical and Biological Engineering Institute of Chemical Processes Institute of Engineering Research Seoul National University Seoul Republic of Korea

**Keywords:** all‐solid‐state batteries, engineering strategies, mechano‐electrochemistry, sulfide‐based solid electrolytes, transition‐metal layered oxides

## Abstract

By replacing flammable organic liquid electrolytes with rigid solid ones, all‐solid‐state batteries (ASSBs) promise higher pack‐level energy density and improved thermal safety than conventional lithium‐ion batteries (LIBs). However, their rigidity accompanies elevated significance of mechanical solid‐solid contact on charge‐transport interfaces during electrochemical operation of cells, which complicates accurate failure diagnosis and obscures rational design rules. This review integrates current understanding of reaction mechanisms and failure pathways of sulfide‐based ASSBs, mapping the critical conduction networks, including intra‐/inter‐CAM transport, CAM|SE interfaces and transport among SE particles in layered oxide composite cathodes and how each is disrupted by electro‐chemo‐mechanical processes during operation. Beyond cathode volume change during charge and discharge, we highlight degradation of sulfide SEs on cathode active material and carbon surfaces and its direct contribution to contact loss and resistance growth, followed by engineering strategies that raise tolerance to stress accumulation and sustain co‐percolation of electronic and ionic transport through cathode morphology/composition control, SE interfacial modification, particle‐size engineering, and pressure management. Although our emphasis is on composite cathodes, the design principles extend to full‐cell architecture and manufacturing, guiding the development of safe, high‐energy‐density ASSBs capable of stable operation at practical low‐pressure conditions.

## Graphical abstract

This review provides an integrated framework for achieving superior electrochemical performance in sulfide‐based all‐solid‐state batteries. It first delineates mechano‐electrochemical failure modes of cathode active materials and solid electrolytes, then outlines engineering principles for particle morphology, electronic and ionic conduction, and interfacial stability. Electrode‐ and cell‐level optimization, including high‐density designs and external pressure control, is also discussed.

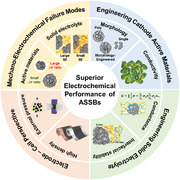

## 1 Introduction

The transition from conventional liquid electrolyte‐based lithium‐ion batteries (LIBs) to all‐solid‐state batteries (ASSBs) is driven by the pursuit of both higher energy density and enhanced safety [1, 2, 3, 4, 5]. Despite their maturity and widespread deployment, LIBs increasingly face intrinsic performance constraints that limit further improvements in energy density for next‐generation applications. By replacing liquid electrolytes (LEs) with solid counterparts, ASSBs can address many of these challenges while enabling new cell architectures and performance enhancements [6, 7, 8]. The adoption of Li metal anodes and bipolar stacking with minimized inactive components is expected to further boost the energy density of ASSBs. Moreover, ceramic‐based ASSBs exhibit reduced volatility compared to liquid systems, thereby mitigating thermal hazards even at high active material loadings [9].

Unlike LIBs, in which the fluidity of the liquid electrolytes enables a uniformly wetted pore network, ASSBs rely on solid‐solid interfaces for charge‐transport, complicating the fabrication of operable cells. While sulfide‐based solid electrolytes partially alleviate this issue with their superior deformability and high ionic conductivity [10, 11], they still require kinetically favorable operation conditions, such as elevated temperature and high operating pressure, to deliver electrochemical performances comparable to that of state‐of‐the‐art LIBs [12, 13, 14]. Therefore, in order to bridge the gap between laboratory demonstrations and practical applications, the working mechanisms and degradation modes of ASSBs need to be scrutinized with a perspective distinct from that used to understand LIBs.

In this regard, this review begins by comparing the electrochemical reactions and degradation mechanisms of LIBs and ASSBs, emphasizing the mechano‐electrochemical characteristics of sulfide‐based composite cathodes. Since all constituents are solid, ASSB electrodes must be designed to ensure continuous electronic and ionic percolation from the initial fabrication stage. During battery operation, beyond the dimensional changes of active materials that contribute significantly to electro‐chemo‐mechanical degradation, [15] recent studies have identified solid electrolyte decomposition as a critical, coupled failure pathway [16, 17]. Achieving high ASSB performance, therefore, depends on constructing well‐percolated electrodes that retain mechanical integrity under operational stress. In addition. engineering strategies to mitigate mechano‐electrochemical challenges through cathode active material (CAM) design and electrolyte modification are systematically summarized. From a manufacturing perspective, we also discuss the scalable fabrication of sheet‐type ASSBs, including powder mixing, binder selection, and the feasibility of thick‐electrode architectures, with particular attention to whether their rate‐limiting factors arise from electronic or ionic transport. Although applying high external pressure can temporarily suppress mechanical degradation, practical operation requires stack pressures limited to a few MPa to meet end‐user demands [18, 19]. Accordingly, design strategies for practical cell‐scale operation under low pressure are discussed in detail. Overall, this review establishes a mechanistic framework linking percolation, interfacial chemistry, and mechanical stress evolution to measurable performance, providing guidance for electrode design and large‐scale implementation of ASSBs.

## 2 Understanding Mechano‐Electrochemistry in All‐Solid‐State Batteries

### 2.1 Comparing the Operating Rules and Failure Modes of LIBs and ASSBs

**FIGURE 1:**
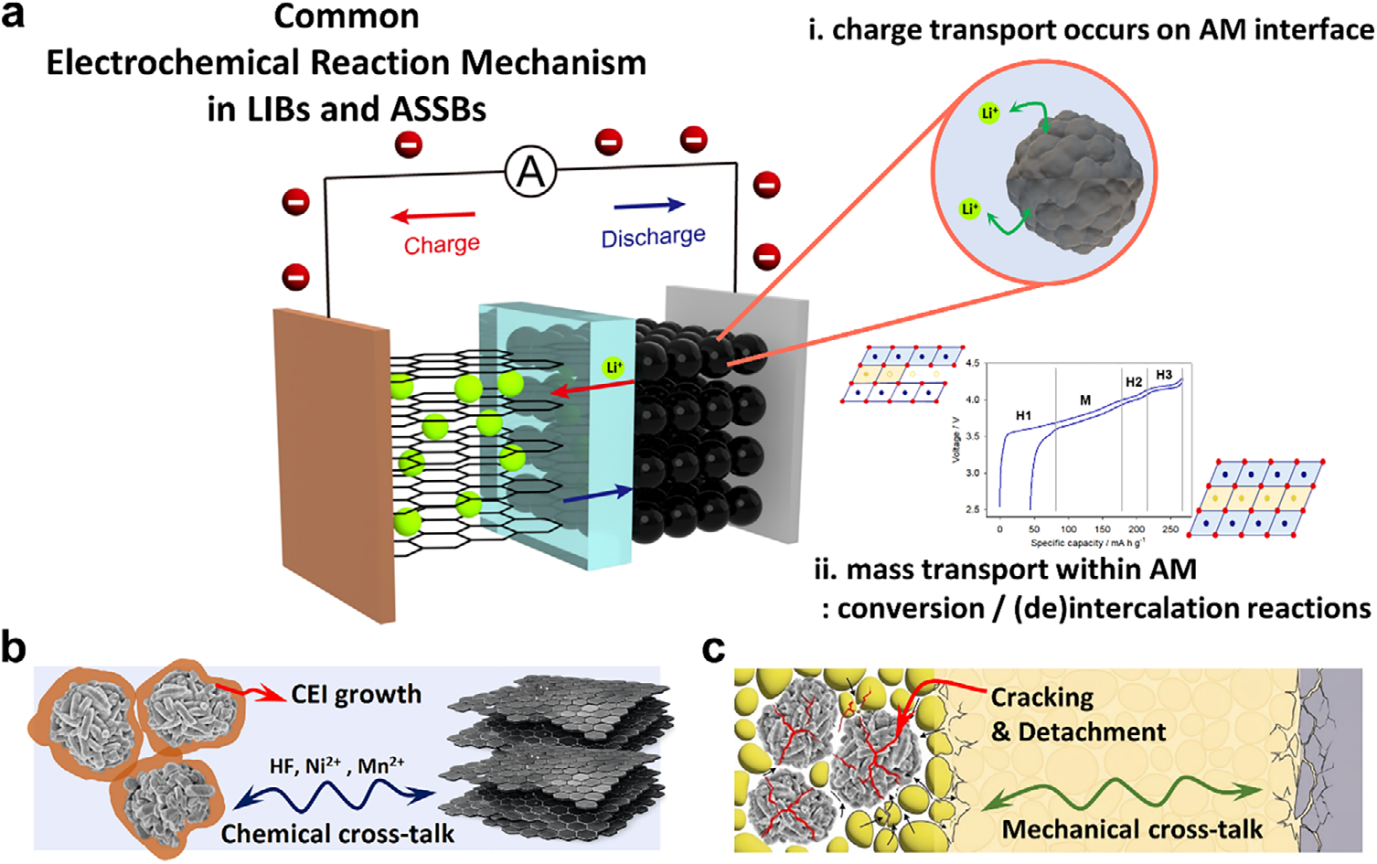
Schematic illustration of (a) common electrochemical reaction mechanisms in LIBs and ASSBs, and their distinct failure modes in (b) LIBs and (c) ASSBs.

Although LIBs and ASSBs share the same fundamental electrochemical principles, they differ markedly in their reaction pathways and dominant degradation modes (Figure 1). As depicted in Figure 1a, charge transfer reactions occur at the interface between active materials (AMs) and electrolytes under an applied current or voltage bias, while the AMs undergo mass transfer reactions through conversion or (de)intercalation chemistries. These reactions are accompanied by changes in particle volume as well as in ionic and electronic conductivity, features that are generally common to active materials for both systems. The evolution of electronic and ionic conduction networks within the electrode then determines the capacity retention over subsequent cycles. However, the degradation modes of LIBs and ASSBs are distinct due to the different fluidities of liquid and solid electrolytes. In LIBs, the fluidity of the LE helps to mitigate mechanical disconnection and suppress abrupt capacity loss caused by dead‐volume formation, whereas exposing fresh AM surfaces accelerates electrolyte decomposition (Figure 1b) [20, 21, 22, 23]. Over repeated cycling, the progressive exposure of new AM surfaces triggers continuous side reactions with the LE, leading to the accumulation of a resistive cathode–electrolyte interphase (CEI) and the gradual depletion of both AM and LE [24, 25, 26]. Moreover, mobile decomposition products, such as dissolved transition‐metal ions, migrate between the cathode and the anode, inducing additional degradation through so‐called cross‐talk phenomena [27, 28, 29]. More specifically, acidic species generated by electrolyte decomposition, such as HF, promote transition‐metal dissolution at the cathode surface, and the dissolved metal ions migrate to the anode surface, where they deposit, forming a solid‐electrolyte interface (SEI) and catalyze further electrolyte reduction. This cathode‐to‐anode chemical shuttle accelerates SEI thickening and increases interfacial resistance along with the consumption of the cyclable Li reservoir, thereby coupling cathode instability directly to anode aging. Consequently, most LIB design strategies focus on suppressing electrolyte consumption and interphase thickening to preserve energy density during cycling, since percolation pathways generally remain intact despite microstructural rearrangements until the eventual mechanical failure of cell components [30, 31]. For ASSBs, constructing well‐percolated electronic and ionic conduction pathways within the pristine composite electrode is closely related to cell performance, as all charge transport occurs through solid–solid contacts (Figure 1c). For example, ASSBs frequently experience abrupt capacity loss within the initial a few cycles even when chemical degradation of the interfaces are not severe, which is due to mechanical detachment between particles that disrupts these pathways [32]. In addition, while the rigidity of SEs can inhibit *‘chemical cross‐talk’* in LIBs, it can introduce the risk of *‘mechanical cross‐talk’*, as the volume changes from one electrode can be transmitted through the load‐bearing solid separator to the counter electrode [33, 34]. Furthermore, although sulfide solid electrolytes were initially expected to be highly safe, a growing body of evidence showing that composite cathodes can exhibit thermal instability raises concerns about their thermal stability with layered oxide cathode materials under practical operating conditions [35, 36]. What appeared to be a straightforward replacement of a liquid electrolyte with a solid one therefore necessitates fundamentally different design strategies: LIBs and ASSBs respond distinctly ‒ both mechanically and chemically ‒ to the electrochemical reactions of active materials, and thus their performance optimization requires divergent approaches.

### 2.2 Percolation‐Guided Design of Composite Cathodes for Sulfide‐Based ASSBs

**FIGURE 2:**
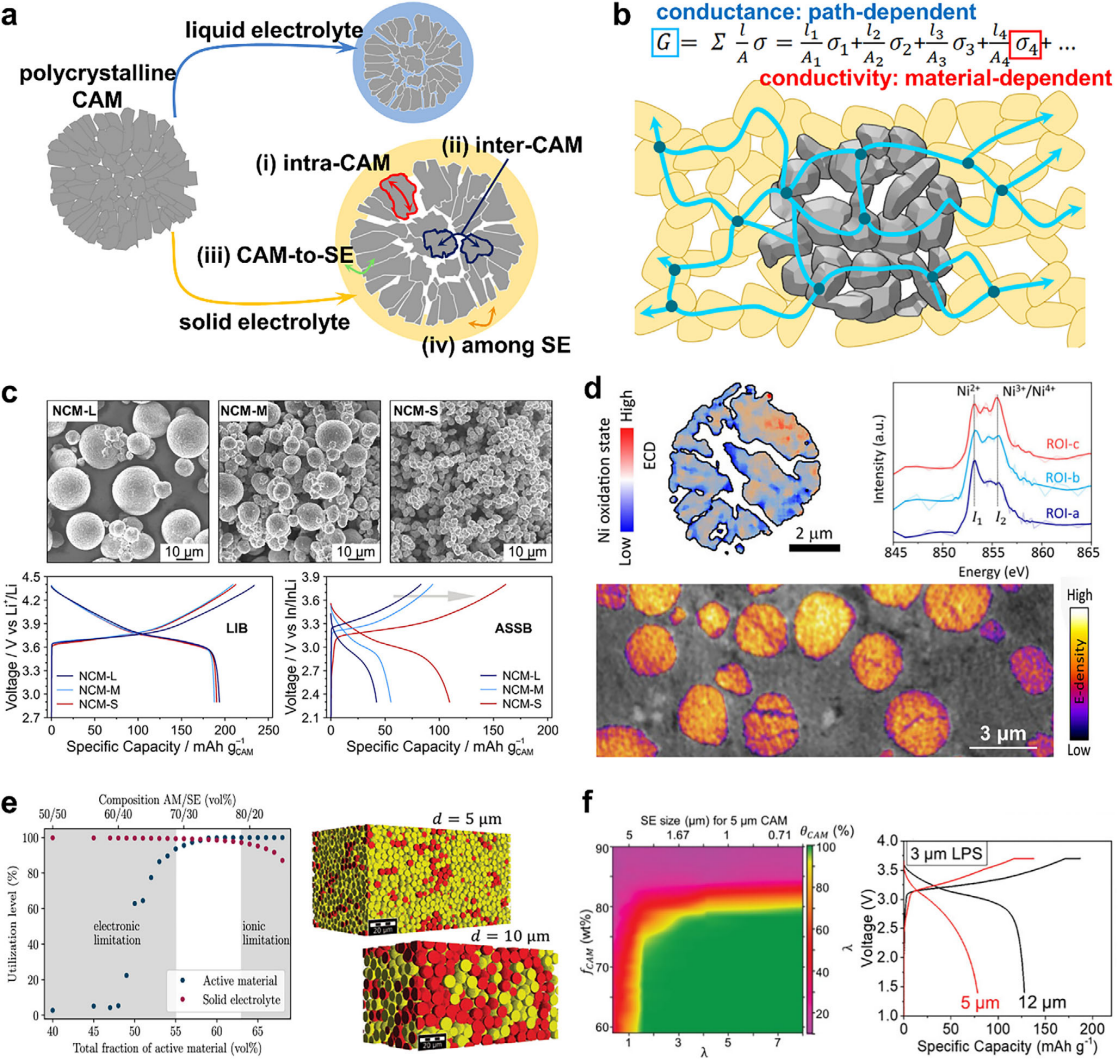
(a) Schematic of critical charge transport interfaces in LIBs and ASSBs. (b) Schematic illustration explaining the concept of conductivity and conductance within composite cathode as a degradation parameter for all‐solid‐state batteries. (c) First‐cycle voltage profiles of NCM with varying secondary‐particle sizes in LIBs versus ASSBs. Reproduced with permission [39]. Copyright 2018, American Chemical Society. (d) Intra‐CAM SOC heterogeneity probed by STXM (top) and inter‐CAM SOC heterogeneity probed by hard X‐ray phase‐contrast holotomography (bottom). Reproduced with permission [42]. Copyright 2025, Wiley‐VCH. (e) CAM‐SE particle distribution within composite cathode with varying solid electrolyte particle size, and modeling of electronic and ionic utilization rate as a function of CAM/SE composition. Reproduced with permission [45]. Copyright 2019, American Chemical Society. (f) Modeling of the cathode utilization (θ_CAM_) as a function of size ratio λ (D_CAM_/D_SE_) and cathode weight fraction (*f*_CAM_), and first‐cycle voltage profiles of 5 and 12 µm NCM using 3 µm LPS as the catholyte. Reproduced with permission [46]. Copyright 2020, Wiley‐VCH.

Figure 2a compares the conduction pathways in composite cathodes for LIBs and ASSBs that are composed of polycrystalline cathode active material (CAM) particles. The primary conduction pathways can be classified as follows: (i) intra‐CAM, occurring within a primary particle of the CAM; (ii) inter‐CAM, between primary particles within the same secondary particle; (iii) CAM‐to‐electrolyte, across the interface between a CAM surface and the electrolyte; and (iv) electrolyte transport, along the LE or through SE particles. In LIBs, CAM is uniformly wetted with LE because LE fills intergranular pores, thereby enlarging the effective surface area for (iii) CAM‐to‐LE conduction and lowering the barrier for (ii) inter‐CAM charge transfer. In contrast, achieving comparable wetting in ASSBs is inherently challenging due to the solid nature of SEs. Although sulfide SEs possess relatively low Young's modulus (tens of GPa) and high ductility, which partially mitigate (iii) CAM‐to‐SE interfacial resistance and improve (iv) transport through SE particles compared with oxide or halide SEs, a significant disparity remains between the wetting behaviors of LEs and SEs [37, 38]. While uniform wetting achieved by liquid electrolytes in LIBs minimizes the concern regarding the connectivity of charge transport pathways, rigid solid‐solid contact in ASSBs raises the issue whether the mechanical contact is actually achieved in ASSBs. For this reason, the concept of “*conductance*”, which accounts for both “*conductivity*”—the intrinsic feasibility of charge transport within a particle—and the actual physical length of charge transport pathway, which is determined by mechanical connectivity of the particles, is more appropriate as a degradation parameter than “conductivity” alone, especially when analyzing ASSB failure mechanisms (Figure 2b). Even when the intrinsic conductivities of electrode constituents are high, ASSBs can still suffer from a decrease in overall electrode‐level conductance if mechanical factors reduce the effective interfacial contact area between particles or elongate the effective charge transport length. Specifically, the particle size or volume ratio and cycling‐induced particle morphology changes can alter solid‐solid contact, thereby diminishing the effective contact area and complicating Li^+^ conduction pathways within the electrode for electrochemical reactions. As a result, the macroscopic conductance of the electrode is a critical parameter that measures the degree of failure in all‐solid‐state batteries.

The concept validity of “conductance” and “conductivity” is illustrated by the distinct dependence of electrochemical performance on CAM secondary particle size in LIBs versus ASSBs [39, 40]. As illustrated in Figure 2c, the electrochemical performance of ASSBs deteriorates much more greatly with increasing CAM secondary particle size than in conventional LIBs. ASSBs employing NCM with secondary particles larger than ca. 5 µm deliver poor charge–discharge capacity compared to those with small particles. This is attributed to the formation of electrochemically inactive dead volumes, corroborated by state‐of‐charge (SOC) heterogeneity between inter‐ and intra‐CAM regions observed via XRD, X‐ray Absorption Near Edge Structure (XANES) and scanning transmission X‐ray microscopy (STXM) analyses (Figure 2d) [41, 42, 43]. These findings highlight that the difference in electrolyte fluidity fundamentally alters the wetting behavior of polycrystalline CAMs, intricating the effective definition of the intra‐CAM Li^+^ diffusion length, whether by the ‘secondary’ or the ‘primary’ particle size in ASSBs.

At the electrode level, percolation‐theory models based on close‐packing assumptions have been employed to establish quantitative design rules for maximizing CAM utilization, providing criteria for the co‐percolation of electronic and ionic networks as functions of composition and particle size [44, 45, 46, 47]. Bielefeld et al. used computational modeling to investigate how the CAM/SE composition and CAM secondary particle sizes influence the formation of electronically and ionically percolating clusters at a fixed SE particle size (Figure 2e) [45]. In their model, carbon additives were excluded; thus, electronic pathways arose solely from CAM–CAM contacts, while ionic pathways were formed through SE–SE contacts. The simulations revealed an optimal CAM/SE volume fraction between 70/30 and 80/20. Below this range, electronic connectivity collapsed sharply, whereas above it, ionic connectivity declined more gradually. Increasing the CAM particle size shifted this optimum toward higher CAM fractions, compensating for the reduced electronically active interfacial area caused by the decreasing surface‐to‐volume ratio. For electrodes where electronic conduction is ensured by carbon additives, Shi et al. introduced a dimensionless size ratio, λ = D_CAM_ / D_SE_, as a critical parameter governing CAM utilization in electrodes (Figure 2f) [46]. Using NCM523 and 75Li_2_S‐25P_2_S_5_ as a model system, their simulations suggested that full CAM utilization requires λ > 1.67. However, even at larger λ values, complete utilization was not achieved once the CAM content exceeded 80 wt.%. These computational results were corroborated by experimental observations across varied CAM and SE particle sizes, demonstrating that the capacity delivered during initial cycles depends primarily on the relative particle‐size ratio rather than on their absolute dimensions.

While these studies provide essential guidance for ASSB electrode design, they primarily address (iii) CAM‐to‐SE interfacial transport and (iv) ionic percolation among SE particles, often under the simplifying assumption that point‐to‐point particle contact is sufficient for complete electrochemical utilization. Such assumptions hold most accurately under low‐rate operation and during the initial cycles, when the complexities of (i) intra‐ and (ii) inter‐CAM kinetics can be reasonably neglected. However, electrochemical cycling inevitably induces CAM volume fluctuations and parasitic reactions that dynamically modify both the effective interfacial area for charge‐transfer and the intrinsic conductivity of the interphases. In the subsequent sections, we examine the fundamental origins of ASSB degradation and outline corresponding mitigation strategies, focusing on how these processes collectively exacerbate electro‐chemo‐mechanical rupture of key conduction pathways. Taken together, the pathway analysis and percolation criteria discussed above indicate that electrode utilization is not dictated solely by static geometry but evolves continuously with cycling as contact integrity, interfacial area, and phase conductivities change. Therefore, effective ASSB design must integrate static co‐percolation thresholds with time‐dependent constraints arising from side reactions and strain evolution. We now turn to the two dominant degradation axes: Section 3 addresses cathode‐volume‐change‐induced contact loss and void formation, while Section 4 explores sulfide solid electrolyte decomposition leading to ion‐blocking interphase formation.

## 3 Mechano‐Electrochemical Failure Modes of Cathode Active Materials and Engineering Strategies in All‐Solid‐State Batteries

Lithium (de)intercalation in active materials induces dimensional changes that serve as a primary cause of particle fracture and void formation, disrupting the conduction pathways connecting cathode particles. Because of the intrinsic rigidity of solid electrolytes, such mechanical contact loss within the composite cathode is difficult to recover, leading to irreversible deformation of the pre‐existing percolation network. Strategies to preserve electronic and ionic connectivity through engineering of cathode active materials can be broadly categorized into three complementary approaches: (i) compositional tuning, aimed at minimizing cycling‐induced volume fluctuations, (ii) morphological design, spanning from primary grains to secondary particle architectures to enhance inter‐CAM mechanical integrity, and (iii) intra‐CAM transport engineering, through targeted doping and grain boundary modification to improve the intrinsic Li^+^ and electronic conductivities.

### 3.1 Electro‐Chemo‐Mechanical Degradation Driven by CAM Volume Change

**FIGURE 3:**
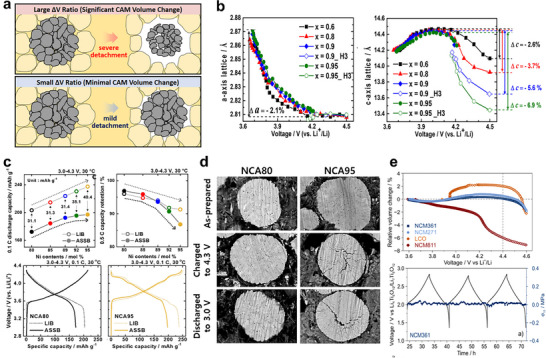
(a) Schematic illustration showing how the cathode volume‐change ratio derives electro‐chemo‐mechanical failure in ASSBs. (b) Evolution of the *a‐* and *c‐*axis parameters of Li[Ni_x_Co_y_Mn_1‐x‐y_]O_2_ during charge as a function of cell voltage. Reproduced with permission [53]. Copyright 2018, American Chemical Society. (c) Electrochemical performance of NCA cathodes with varying Ni content in LIBs versus ASSBs and (d) cross sectional SEM images of NCA80 and NCA95 in ASSBs in the pristine state, after charging to 4.3 V, and after discharging to 3.0 V (vs. Li/Li^+^). Reproduced with permission [55]. Copyright 2023, Royal Society of Chemistry. (e) Relative volume change ratios of layered transition‐metal oxide cathodes and in situ differential electrochemical pressiometry (DEP) result for NCM361 in ASSBs. Reproduced with permission [60]. Copyright 2020, American Chemical Society.

As demonstrated in Figure 3a, dimensional changes of CAMs during cycling are a major source of mechanical failure in ASSBs. Layered transition‐metal oxides, such as NCM and NCA, undergo pronounced anisotropic lattice evolution upon (de)lithiation [48, 49]. During delithiation, the in‐plane *a‐* and *b*‐axes lattice parameters decrease approximately linearly with Li stoichiometry, reflecting the strengthened TM‒O bonds as transition‐metals become oxidized. In contrast, the interlayer *c*‐axis lattice parameter initially expands because Li^+^ extraction reduces electrostatic screening and enhances O–O repulsion between adjacent oxygen slabs. Beyond a critical state of charge (SOC), typically above 80% Li extraction, the H2–H3 phase transition triggers an abrupt *c*‐axis contraction, which is commonly referred to as *c*‐axis collapse, accompanied by slab gliding and densification of the inter‐slab spacing. This collapse is particularly pronounced in Ni‐rich layered oxides, which permit deeper delithiation but suffer from weaker interlayer stabilization (Figure 3b) [50, 51, 52, 53, 54]. In LIBs, one of the key challenges in employing Ni‐rich cathodes is that their significant volume fluctuations expose fresh interparticle surfaces, promoting LE decomposition and leading to thermodynamic instability at high SOC. In ASSBs, however, such lattice shrinkage manifests differently: it induces mechanical detachment along the CAM–SE interfaces and inter‐CAM cracking, especially for large polycrystalline architectures with randomly‐oriented primary particles.

Yu et al. reported that the effective utilization of Ni‐rich layered oxide materials in ASSBs is significantly hindered by their dimensional changes from the very first cycle (Figure 3c) [55]. Electrochemical analyses of 10 µm‐sized polycrystalline NCA particles with varying Ni contents above 0.8, examined in both conventional LIBs and Li_6_PS_5_Cl (LPSCl)‐based ASSBs, revealed that although the initial charge capacity increased with higher Ni content in both systems, which is consistent with their theoretical capacity, the initial coulombic efficiency and capacity retention declined, with a much more pronounced deterioration observed in ASSBs. This indicates that dimensional shrinkage of Ni‐rich layered oxide cathodes leads to particle detachment (Figure 3d), and that ASSBs are considerably more susceptible to such electro‐chemo‐mechanical failure, even at low C‐rates and during early cycling. Further analyses using galvanostatic intermittent titration technique (GITT) and electrochemical impedance spectroscopy (EIS) confirmed that charge‐transfer kinetics are substantially slower in ASSBs, particularly near the end of charge. Given that NCA volume shrinkage intensifies at high SOC, these findings suggest that particle disintegration during delithiation disrupts charge‐transfer pathways in ASSBs, whereas in LIBs, the fluidic nature of the liquid electrolyte partially restores interfacial contact. Overall, the study underscores that the pronounced lattice‐volume change ratio of Ni‐rich layered oxides can fundamentally limit their viability as high–energy‐density cathodes in ASSBs.

One strategy to mitigate the dimensional changes of CAMs is to operate them at lower charge cut‐off voltages, as the *c*‐axis collapse is more pronounced at high SOC. This approach has proven effective in enhancing the cycling stability of Ni‐rich cathodes in LIBs [51, 56, 57] and is expected to be equally, if not more, beneficial in ASSBs, [58, 59] albeit at the expense of reduced energy density. An alternative strategy involves tuning the transition‐metal composition of layered oxides to minimize their overall volume‐change ratio. Strauss et al. investigated the molar volume evolution during delithiation of NCMs with varying Co/(Co+Ni) ratios and demonstrated that suppressing lattice volume changes at the atomic level leads to smaller pressure fluctuations in ASSBs during operation (Figure 3e) [60]. In situ XRD measurements of NCM271 and NCM361 during the first cycle in LIBs revealed that anisotropic variations along the *a*‐ and *c*‐axes can offset each other, resulting in negligible net changes in unit‐cell volume. Although Ni‐poor cathodes are less attractive for practical applications due to their lower specific capacities, the findings by Yu et al. and Strauss et al. collectively suggest that incorporating dopants capable of suppressing *c*‐axis collapse during the H2–H3 transition can significantly enhance ASSB performance. Accordingly, the roles of common transition‐metal‐site dopants (e.g., Zr, Ti, Nb, W, and others) warrant re‐evaluation from an electro‐chemo‐mechanical perspective in the context of ASSBs [61, 62]. In contrast, Li‐site modulation requires additional caution, as the ionic conductivity and defect behavior of such heteroatoms in sulfide‐based solid electrolytes remain poorly understood [63, 64, 65].

### 3.2 Morphological Engineering to Increase Mechanical Integrity between CAM Particles

**FIGURE 4:**
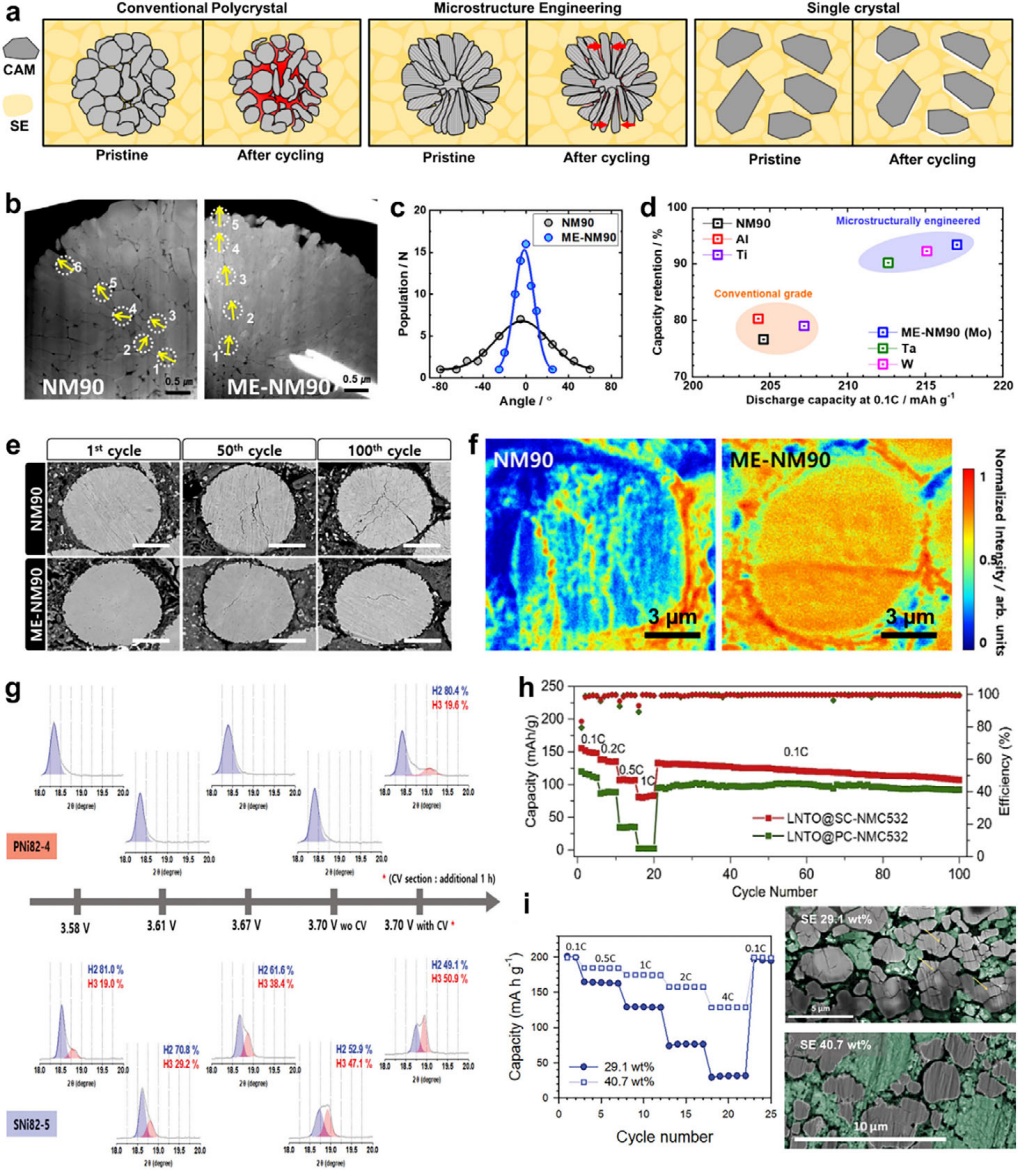
(a) Schematic illustration of morphological engineering strategies of CAMs to mitigate electro‐chemo‐mechanical failure in ASSBs. (b) Cross sectional TEM images, (c) crystallographic orientations of primary particles in bare and morphology‐engineered (ME) NM90 and (d) electrochemical performance of bare and ME‐NM90 in ASSBs. (e) Cross sectional SEM images of bare and ME‐NM90 in the pristine state, retrieved at fully charged states after 50 cycles and after 100 cycles in ASSBs. (f) ToF‐SIMS Li^+^ mapping images of bare and ME‐NM90 retrieved in the discharged state after 100 cycles in ASSBs. (b–f) Reproduced with permission [71]. Copyright 2025, American Chemical Society. (g) Ex situ XRD patterns of polycrystalline and single‐crystalline NCM composite cathodes retrieved at different states of charge in ASSBs. Reproduced with permission [79]. Copyright 2024, Wiley‐VCH. (h) Rate and cycle performance of polycrystalline and single‐crystalline NCM (2 – 5 µm) in ASSBs. Reproduced with permission [82]. Copyright 2020, Elsevier. (i) Rate performance of single‐crystalline NCA in halide‐based ASSBs and cross sectional SEM images of composite cathodes retrieved after cycling, for varying halide solid electrolyte weight fractions. Reproduced with permission [84]. Copyright 2021, Wiley‐VCH.

While the volume change of CAM particles is a primary cause of conduction‐pathway loss around active materials, Ni‐rich compositions remain favored over Ni‐poor counterparts due to their intrinsically higher energy density, despite the fact that they exhibit larger dimensional variations. Contact‐loss between particles can be considered as a combined effect of the lattice volume change ratio and particle dimensions, in analogy with the relationship between conductivity and conductance. On this basis, strengthening the mechanical integrity of three CAM‐related conduction pathways, such as intra‐CAM, inter‐CAM, and CAM‐to‐SE, by morphological engineering of CAM particles offers a relatively facile strategy to enhance ASSB performance (Figure 4a). Reducing the secondary particle size of CAMs shortens CAM|SE detachment length upon CAM volume change, increases the CAM‐SE interfacial area, and also shortens the intra‐CAM Li^+^ diffusion length, all of which attributes to improved electrochemical performance, as discussed in Section 2.2. Since it can alleviate the impact of contact loss, such strategies are particularly effective for CAMs exhibiting large dimensional changes and intrinsically poor kinetic limitations [66, 67, 68].

For polycrystalline CAMs in ASSBs, tailoring the morphology of primary particles to enhance inter‐CAM Li^+^ conduction has emerged as a promising design strategy [69, 70, 71, 72]. In contrast to randomly oriented polycrystalline CAMs, columnar NCM aggregates consist of primary grains elongated within the ab plane and shortened along the *c*‐axis, such that the edge planes predominantly form the surface of each secondary particle (Figure 4b,c). Ni‐rich cathodes undergo pronounced lattice shrinkage along the c‐axis during delithiation. Consequently, this aligned microstructure effectively relieves mechanical strain and mitigates interparticle cracking within secondary particles, thereby preserving continuous Li^+^ conduction pathways along the ab plane [72, 73]. Sun's group systematically investigated this approach using Ni‐rich materials (Ni > 0.8) with secondary‐particle sizes up to 10 µm and varying degrees of primary‐particle orientation [69, 70, 71]. They also evaluated the influence of surface‐protective coatings to isolate the role of CAM‐SE interfacial conduction, which is discussed in greater detail in Section 4. Remarkably, morphology engineering substantially improved both initial capacity and capacity retention (Figure 4d). Cross sectional SEM images confirmed a marked reduction in inter‐CAM cracking for morphology‐engineered cathodes compared to randomly oriented counterparts (Figure 4e), which in turn facilitated more complete Li^+^ reinsertion into the bulk during discharge (Figure 4f). These findings indicate that, as the secondary particle size increases, inter‐CAM mechanical failure induced by electrochemical lattice volume changes becomes a more dominant degradation factor in ASSBs than interfacial CAM|SE contact loss. Although morphological engineering of primary particle orientation effectively enhances the cycle performance of large, Ni‐rich cathodes, such columnar grains are generally smaller and introduce a higher density of grain boundaries within each secondary particle. Consequently, their electrochemical robustness under crack‐prone conditions, such as high C‐rates or elevated‐temperature calendar aging, requires further evaluation.

Enhancing charge‐transfer homogeneity and reducing tortuosity within cathode materials through single‐crystallization has proven effective for improving the electrochemical performance of ASSBs [74, 75]. While polycrystalline CAMs are prone to inter‐CAM mechanical disintegration, single‐crystalline counterparts exhibit significantly greater structural robustness [76, 77, 78]. Figure 4g presents ex situ XRD patterns of polycrystalline and single‐crystalline NCMs in ASSBs retrieved at different cut‐off voltages, as reported by Hong et al. [79]. The results show that the H2–H3 phase transition is clearly observed in single crystals at ∼4.2 V (vs. Li/Li^+^), but only marginally detected in polycrystals even after a constant‐voltage hold at ∼4.3 V (vs. Li/Li^+^), emphasizing the superior mechanical integrity of single crystals against interparticle detachment during charging. This stability translates into improved cycling and rate performance.

In LIBs, the enhanced mechanical integrity of single crystals is typically achieved at the cost of larger primary particle sizes and thus longer intra‐CAM Li^+^ diffusion paths, leading to slightly inferior rate capability compared with polycrystalline counterparts [80, 81]. In contrast, when overall particle sizes are comparable, single‐crystalline CAMs are known to outperform polycrystals in terms of both rate and cycle performance in ASSBs (Figure 4h) [79, 82, 83]. However, the CAM/SE ratio generally requires re‐optimization upon single‐crystallization, as the effective CAM|SE interfacial area changes. Han et al. reported contrasting behaviors depending on the electrolyte type: single‐crystalline cathodes outperformed polycrystalline ones in sulfide‐based ASSBs, whereas the opposite trend was observed in halide‐based systems (Figure 4i) [84]. Considering the higher density of halide electrolytes, increasing their weight fraction in composite cathodes improved the discharge capacity and cycling stability of single crystals relative to polycrystals.

These results highlight that while reducing CAM particle size can enhance kinetics, it often necessitates rebalancing of the composite formulation, including the CAM/SE ratio and conductive additive content, to sustain optimal electronic and ionic percolation. Consequently, improvements in rate capability do not always translate into higher overall cell energy density. Moreover, the upper particle‐size limit for full electrochemical utilization of single crystals is expected to be smaller in ASSBs than in LIBs. Simulations and experiments have shown that anisotropic lattice changes of NCM induce more severe cracking for larger primary particles, even within single crystals, leading to more critical failure in ASSBs due to their rigid interfaces [49, 85]. To address this, facet‐engineered single crystals designed to dissipate internal stress and facilitate Li^+^ transport may represent a promising pathway toward mechanically resilient, high‐performance ASSB cathodes [86, 87].

### 3.3 Enhancing Intrinsic Charge Transport in Cathode Active Materials: Bulk/Grain‐Boundary Conductivity and CAM|SE Interfacial Kinetics

**FIGURE 5:**
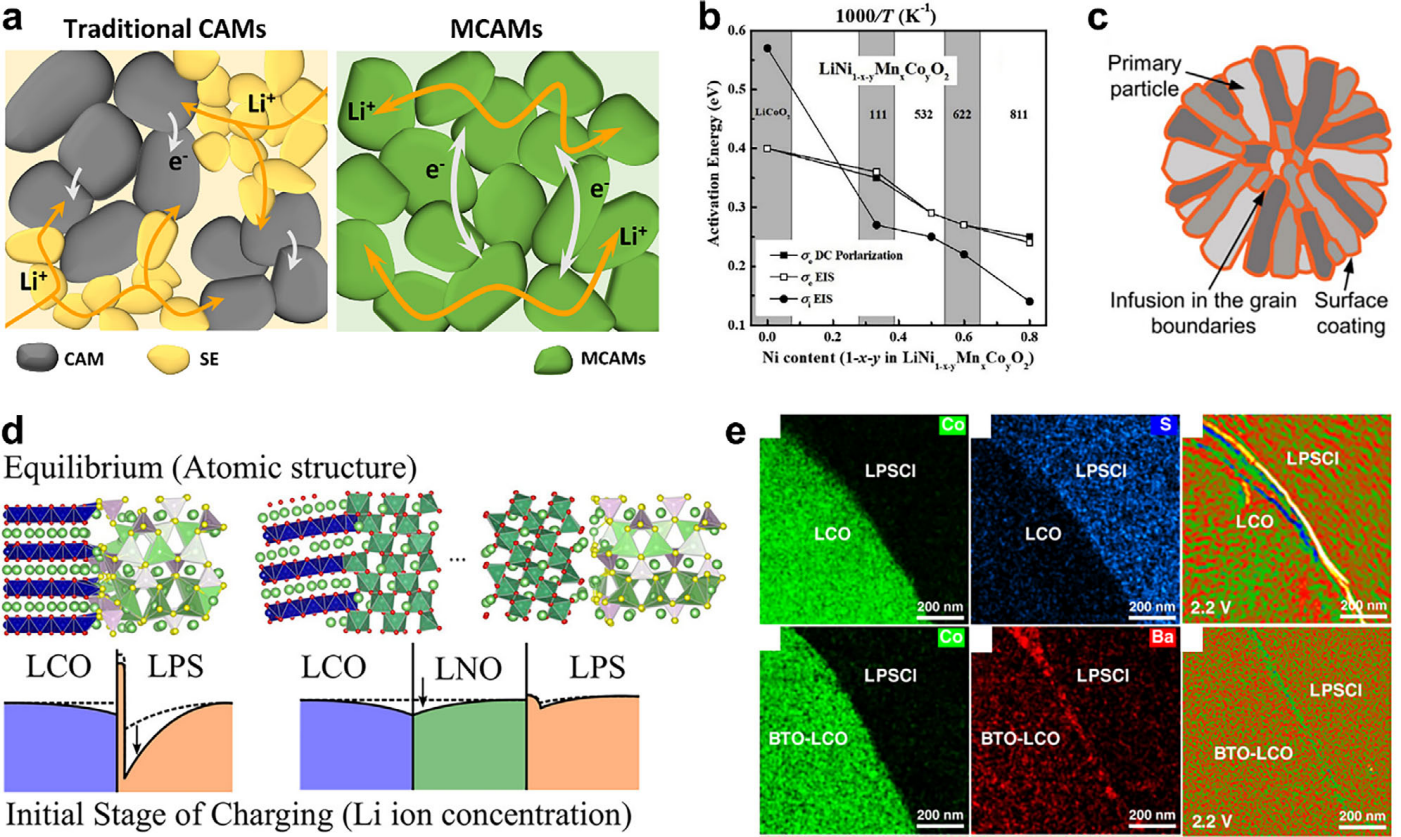
(a) Schematic illustration of conduction pathways within electrodes employing traditional CAMs and MCAMs. (b) Electronic and ionic conductivities of Li[Ni_x_Co_y_Mn_1‐x‐y_]O_2_. Reproduced with permission [95]. Copyright 2018, Elsevier. (c) Schematic illustration of infusion coating on polycrystalline CAMs to enhance ionic transport. Reproduced with permission [97]. Copyright 2022, Wiley‐VCH. (d) Simulated crystal structures and calculated Li^+^ concentration profiles in the space‐charge layer at LCO|LPS and LCO|LiNbO_3_(LNO)|LPS interfaces. Reproduced with permission [104]. Copyright 2020, American Chemical Society. (e) HAADF and in situ DPC‐STEM images of LCO|LPSCl and LCO|BTO|LPSCl, showing mitigation of the space charge layer with a BTO coating on LCO. Reproduced with permission [105]. Copyright 2020, Springer Nature.

Since electronic and ionic conduction pathways in composite cathodes are strongly influenced by the particle size and volumetric ratio between CAMs and SEs, the use of mixed‐conducting active materials (MCAMs), which exhibit both high electronic and ionic conductivity, has been proposed as a promising strategy [88]. As illustrated in Figure 5a, MCAMs enable the design of SE‐free cathodes, eliminating CAM|SE interfacial transport limitations and associated degradation, eventually pushing the electrode‐level energy density closer to the intrinsic energy density of the active material itself. However, the development of materials that combine high ionic and electronic conductivity with electrochemical reversibility and high energy density remains a significant challenge. To date, only a few studies have demonstrated SE‐free cathodes in ASSBs, typically by employing ultrathin electrodes (a few micrometers thick) or operating at elevated temperatures [89, 90, 91, 92]. In practical systems, tuning the transition‐metal composition of commercial layered oxides has proven effective in improving intrinsic charge‐transport kinetics [93, 94, 95, 96]. For NCM and NCA materials, both electronic and ionic conductivities increase with higher Ni content, as the enlarged *c*‐axis facilitates faster Li^+^ migration (Figure 5b) [95, 96]. However, as discussed in Section 3.1, this improvement comes at the cost of greater dimensional changes and mechanical vulnerability, which compromise cycling stability despite favorable kinetics. When Ni content is fixed (similar lattice‐volume change ratios), further optimizing the transition‐metal composition to enhance charge transport has been shown to yield superior rate performance in ASSBs [93]. In addition, modifying grain boundaries with Li^+^‐conducting phases to enhance conductivity, either through infusion coatings or electrolyte infiltration, has also proven effective in improving electrochemical performance (Figure 5c) [97, 98, 99].

In addition to Li^+^ transport within the CAM bulk, the CAM|SE interface represents a critical bottleneck for ion conduction in composite cathodes [100, 101]. Owing to the mismatch in Li^+^ chemical potential between layered oxide cathodes and solid electrolytes, typically lower in sulfide‐based SEs, a space‐charge layer (SCL) forms at their interface (Figure 5d) [102, 103, 104]. DFT calculations by Haruyama et al. revealed that at the LCO|Li_3_PS_4_ interface, Li^+^ ions migrate from the SE toward the CAM, resulting in Li^+^ accumulation on the CAM side and the formation of a Li^+^‐depleted region within the SE [104]. This SCL acts as a kinetic barrier to Li^+^ migration during charging, particularly in sulfide‐based systems. To mitigate this effect, they proposed introducing a LiNbO_3_ coating on LCO as a buffer layer to facilitate Li^+^ transport by alleviating SCL‐induced concentration polarization. Wang et al. subsequently provided experimental confirmation using high‐angle annular dark‐field scanning transmission electron microscopy (HAADF‐STEM) (Figure 5e), demonstrating that the introduction of a BaTiO_3_ coating layer effectively suppressed positive‐charge accumulation at the LCO|LPSCl interface [105]. These findings highlight that understanding the mechano‐electrochemical behavior of cathode materials alone is insufficient to fully elucidate ASSB degradation. Effective design and optimization require a deeper investigation into the electro‐chemo‐mechanical coupling at the CAM|SE interface, where ionic, electronic, and mechanical processes are intricately interdependent.

## 4 Solid Electrolyte Decomposition as Overlooked Driver of Electro‐Chemo‐Mechanical Failure in All‐Solid‐State Batteries

Since the first discovery of Li_3_PS_4_, compositions and crystal structures of sulfide solid electrolytes have been systematically engineered to enhance Li^+^ conductivity and expand the electrochemical stability window [106, 107, 108]. The electrochemical and chemical stability of SEs against layered oxide cathodes has been extensively investigated, with particular emphasis on identifying the composition of the degraded interphase and its impact on overpotential growth through conductivity loss [109, 110]. Although volume changes in CAMs have traditionally been regarded as the primary cause of mechanical contact loss in ASSBs during cycling, recent studies increasingly highlight the pivotal role of SE interfacial degradation in driving this failure mode [111, 112, 113].

In this section, we discuss the multifaceted role of SEs in ASSB operation and outline strategies to improve performance in three parts: (i) enhancement of ionic conductance through compositional and size engineering, (ii) mitigation of electrochemical and chemical degradation via interfacial and compositional modifications, and (iii) elucidation of the SE's role in electro‐chemo‐mechanical failure and exploration of potential mechanical healing strategies.

### 4.1 Ionic Transport Engineering in Sulfide SEs: Composition and Size

Among ceramic solid electrolytes, sulfide‐based systems have emerged as leading candidates for ASSBs owing to their high ionic conductivities comparable to liquid electrolytes and favorable mechanical properties, attracting substantial attention from both academia and industry. Early studies by Tatsumisago's group in the 2000s demonstrated that the Li_2_S–P_2_S_5_ binary system can achieve conductivities of ∼1 mS cm^‒1^ while retaining sufficient ductility for bulk‐type ASSB fabrication via cold pressing [114, 115]. Subsequently, Kanno's group reported Li_10_GeP_2_S_12_ (LGPS)‐type electrolytes exhibiting room‐temperature conductivities exceeding 10 mS cm^‒1^, igniting intense research into sulfide SEs [116]. The later discovery of argyrodite‐type sulfides (Li_6_PS_5_X, X = Cl, Br, I, and their combinations) by Deiseroth et al. further stimulated industrial interest by offering moderate conductivity and lower material costs through the elimination of expensive elements [117, 118].

**FIGURE 6:**
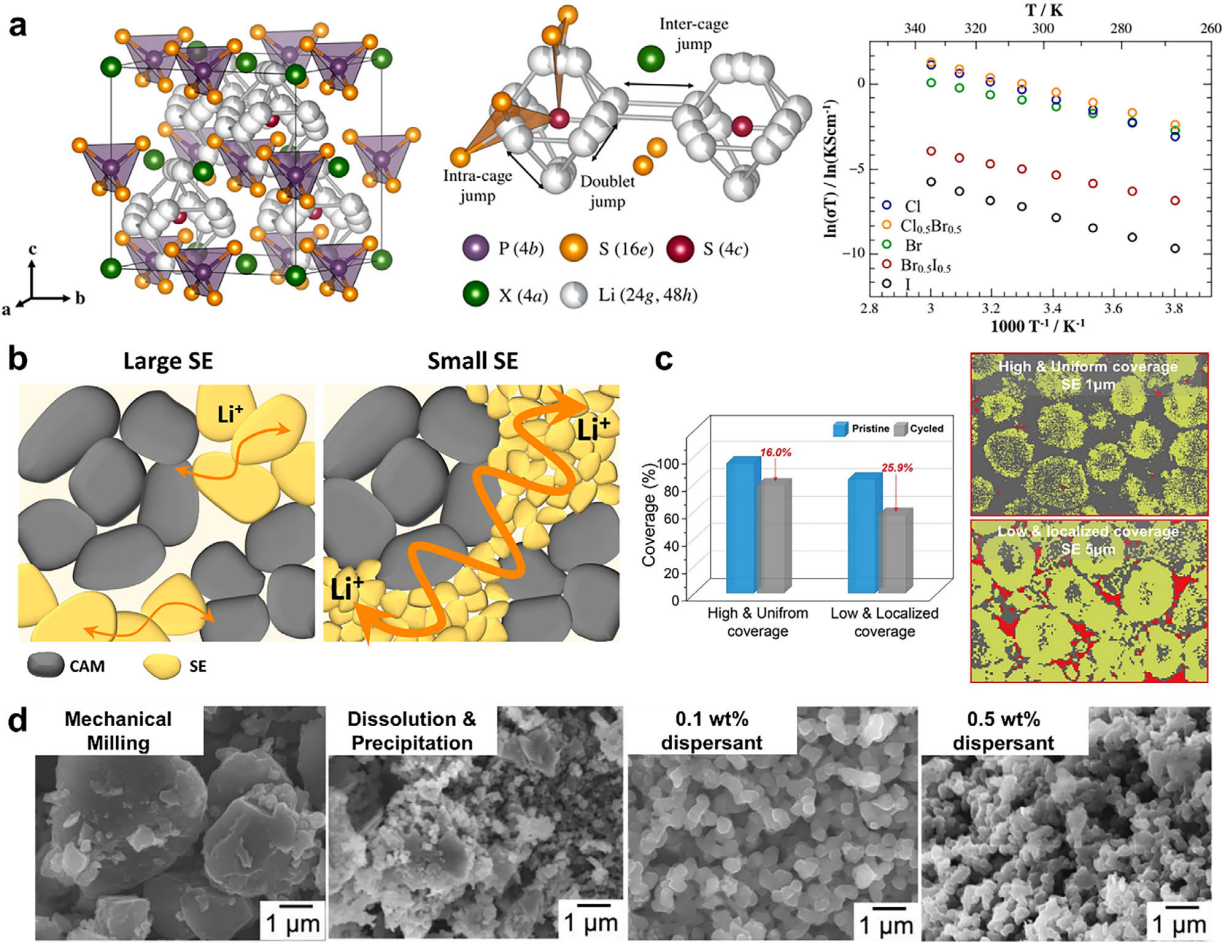
(a) Schematic illustration of argyrodite crystal structure and Li^+^ conductivity of argyrodite‐type Li_6_PS_5_X (X = Cl, Br, I) as a function of halide composition. Reproduced with permission [126]. Copyright 2017, American Chemical Society. (b) Schematic of Li^+^ percolation in composite cathodes with varying SE sizes. (c) Coverage ratio of the surface of cathode particle surface within composite cathode using 1 and 5 µm LPSCl, at pristine state and after cycling. Reproduced with permission [130]. Copyright 2025, Elsevier. (d) Particle‐size distributions and SEM images of LPSCl synthesized by mechanical milling and dissolution—precipitation routes with varying amount of dispersant. Reproduced with permission [139]. Copyright 2018, Elsevier.

Early research on sulfide SEs primarily focused on compositional and structural modifications to enhance Li^+^ conductivity (Figure 6a). A general design principle is that larger and more polarizable ions facilitate Li^+^ transport [119, 120]. For instance, substitutions such as P^5+^ → Sb^5+^, Cl^‒^ → Br^‒^, or S^2‒^ → Se^2‒^ have been shown to increase ionic conductivity [106, 121, 122]. In argyrodite‐type sulfides, the correlation between halide composition and Li^+^ conductivity has been systematically explored [123, 124, 125, 126, 127]. Derived from the Li_7_PS_6_ framework, halide‐containing argyrodites crystallize in a cubic structure where X^‒^ anions occupy the Wyckoff 4a and 4c sites, forming a face‐centered cubic lattice. PS_4_^3‒^ tetrahedra reside at octahedral sites, while free S^2‒^ anions fill half of the tetrahedral sites, enabling Li^+^ diffusion through interconnected cage‐like pathways. The degree of site disorder between X^‒^ and free S^2‒^ anions, which is strongly dependent on halide composition and synthesis conditions, critically influences both Li^+^ conductivity and electrochemical stability. For example, replacing Cl^‒^ with Br^‒^ generally enhances ionic conductivity, whereas substitution with I^‒^ reduces it, despite I^‒^ being larger and more polarizable. This is attributed to limited S^2‒^/I^‒^ site disorder arising from their size mismatch. Additionally, aliovalent doping has been demonstrated to further improve ionic conductivity by introducing Li^+^ vacancies or increasing the population of mobile ions [128, 129].

While early studies primarily focused on compositional engineering of sulfide SEs, the particle‐scale morphology of SEs has proven equally critical for their practical use as catholytes (Figure 6b) [130, 131, 132]. Using smaller SE particles as the catholyte improves the rate and cycling performances of ASSBs because they can more effectively fill interstitial voids between CAM particles, increasing CAM surface coverage and reducing electrode porosity. As shown in Figure 6c, composite cathodes with 5 µm LPSCl exhibited a lower initial CAM surface coverage ratio than those with 1 µm LPSCl, and the coverage decreased more sharply after cycling when larger SE particles were used. This trend suggests that finer SE particles not only maximize the initial CAM‐SE contact area during electrode fabrication, but also better accommodate electrochemically induced micro‐strains, thereby preserving percolation pathways [130]. Recent progress in SE synthesis has therefore shifted toward controlled morphology engineering, evolving from conventional solid‐state mechanical milling to liquid‐mediated synthesis [133, 134, 135, 136, 137, 138, 139]. Since the first report by Liu et al. on the liquid‐phase synthesis of β‐Li_3_PS_4_, [133] various solvent‐based routes have been developed, generally classified into two categories: (i) suspension synthesis, in which precursors are dispersed and mechanically mixed in a liquid medium, and (ii) dissolution–precipitation synthesis, where dissolved precursors are subsequently precipitated as sulfide SEs. Although surface oxidation during exposure to organic solvents remains a persistent challenge, these solvent‐mediated methods, particularly dissolution–precipitation routes, enable the production of uniform, monodisperse SE particles of a few micrometers in size. Importantly, they also offer scalability for large‐scale manufacturing, providing a practical pathway toward high‐performance, processable SE catholytes (Figure 6d) [139].

### 4.2 Electro‐/Chemical Stability of Sulfide SEs with CAM and Carbon Additives

**FIGURE 7:**
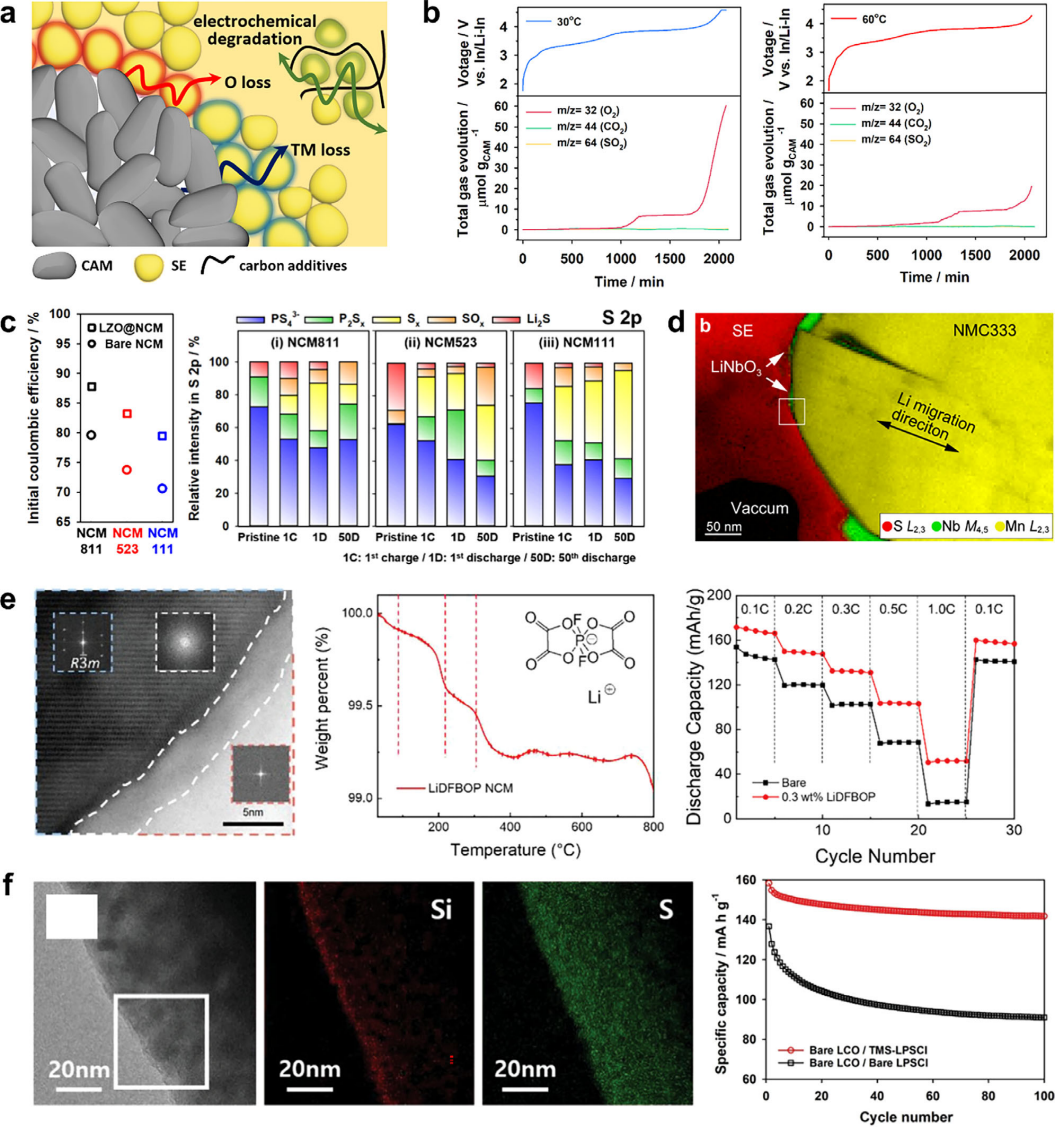
(a) Schematic illustration of electrochemical/chemical degradation pathways of sulfide SEs in composite cathodes employing layered oxide cathodes and carbon additives. (b) Comparison of O_2_ gas evolution quantified by in situ DEMS during cycling of LMRO cathodes at 30°C and 60°C. Reproduced with permission [66]. Copyright 2024, Wiley‐VCH. (c) ICE and XPS results for NCM811, NCM523 and NCM111 in LPSCl‐based ASSBs. Reproduced with permission [146]. Copyright 2025 Wiley‐VCH. (d) Cross sectional STEM‐EELS mapping images of LiNbO_3_‐coated NCM after cycling in a sulfide‐based ASSB. Reproduced with permission [155]. Copyright 2022, American Chemical Society. (e) TEM image, thermogravimetric analysis profile and rate performance of LiDFBOP coated NCM in ASSBs. Reproduced with permission [156]. Copyright 2023, Wiley‐VCH. (f) TEM‐EDS mapping images of LPSCl surface modified with TMS‐SH and its cycle performance with LCO. Reproduced with permission [112]. Copyright 2023, Wiley‐VCH.

The susceptibility of sulfide SEs to oxidation represents a critical challenge in composite cathodes that incorporate layered oxide active materials and carbon additives (Figure 7a) [109]. In LIBs, as the Ni content of layered oxides increases, the thermodynamic instability of lattice oxygen rises sharply, making the cathodes more prone to irreversible O_2_ release and surface reconstruction toward spinel‐ or rock‐salt‐like phases [53, 140]. Sulfide‐based ASSBs have also exhibited similar gas evolution during the charging of layered oxide cathodes, [66, 141, 142] where gaseous O_2_ can permeate through the residual pores in the composite electrode, oxidatively decomposing the SE surface [143]. Chemical side reactions between unstable lattice oxygen in high‐SOC CAMs and sulfide SEs produce interfacial phosphate and sulfate species, with the extent of degradation increasing at elevated temperatures [110, 144, 145]. For instance, Song et al. compared gas evolution in LPSCl‐based ASSBs using Li‐and Mn‐rich layered oxides (LMRO) at 30°C and 60°C [66]. Despite achieving similar charge capacities and thus comparable SOC displayed in Figure 7b, in situ differential electrochemical mass spectrometry (DEMS) detected less O_2_ evolution at 60°C than at 30°C. Time‐of‐flight secondary ion mass spectrometry (ToF‐SIMS) analyses revealed more pronounced oxidative decomposition of the SE at higher temperature, consistent with the explanation that oxygen released from the cathode was consumed by SEs to form phosphate and sulfate species. Consequently, less unreacted O_2_ was detected by DEMS. The extent of chemical reactivity between unstable lattice oxygen from cathodes at high‐SOC and sulfide solid electrolytes has been reported to result in thermal incidents under extreme conditions, which is described with greater detail in Section 6.

The electrochemical and chemical stability between layered oxide cathodes and solid electrolytes strongly depends on the transition‐metal composition of the cathode [146]. In conventional LIBs, transition‐metal dissolution driven by electrolyte acidification is a major cause of capacity fading, particularly in Mn‐rich cathodes [147]. Although such dissolution was initially expected to be mitigated in ASSBs, interdiffusion of transition‐metals from CAMs into sulfide SEs has been observed, forming metal sulfides analogous to the dissolution processes in liquid systems [148, 149, 150]. TEM and micro–XANES analyses have confirmed this phenomenon, especially for Co‐containing cathodes. Park et al. investigated LPSCl interfacial degradation as a function of transition‐metal composition in NCM cathodes under high operating pressure (Figure 7c) [146]. Although the degree of lattice volume change increases with higher Ni content, the initial coulombic efficiency (ICE) in ASSBs followed the trend NCM811> NCM523> NCM111. Spectroscopic analyses revealed that the extent of LPSCl decomposition became more severe with increasing Co content. This behavior was attributed to the higher electronic conductivity of cobalt sulfides relative to nickel or manganese sulfides, which likely accelerates CEI formation. These findings highlight the fundamental differences between liquid and solid electrolyte interfacial chemistries with layered oxide cathodes, and suggest that interfacial side reactions significantly contribute to degradation of sulfide‐based ASSBs, alongside electro‐chemo‐mechanical effects.

Cathode surface coatings such as Li_3_PO_4_, LiNbO_3_ and Li_2_ZrO_3_ are widely employed to mitigate direct contact between charged cathode surfaces and sulfide SEs, thereby enhancing interfacial stability and overall performance [151, 152]. However, various analyses including TEM have revealed that these oxide coatings can partially decompose and exfoliate after cycling, [153, 154, 155] underscoring the need for more resilient surface‐modification strategies to stabilize the CAM|SE interface (Figure 7d). One promising approach involves the use of organic coatings, which exhibit low oxygen‐release tendencies upon decomposition and possess low Young's modulus, providing mechanical compliance during CAM volume changes [156, 157]. Nanometer‐thick organic derivatives of Li salts applied to CAM surfaces have been shown to suppress interfacial resistance growth and improve cycling stability (Figure 7e). A complementary strategy is to enhance the intrinsic oxidative stability of the SE itself, for instance, by replacing P–S bonding with thermodynamically more stable P–O bonding [158, 159, 160]. Zuo et al. further demonstrated that tuning the LPSCl composition can promote gas‐phase removal of interfacial degradation products [161]. Compared with stoichiometric Li_6_PS_5_Cl, Cl‐rich Li_5.5_PS_4.5_Cl_1.5_ generated a greater amount of gaseous species during charging, which alleviated the buildup of solid decomposition products at the interface, reduced interfacial resistance, and improved cycling performance.

Beyond the cathode surface, electrochemical oxidation of sulfide SEs on carbon additives also contributes significantly to degradation [162, 163]. Although sulfide SEs were initially assumed to be stable within the operating voltage range of layered oxides, cyclic voltammetry (CV) and linear sweep voltammetry (LSV) measurements using SE–carbon composite electrodes have revealed an oxidation onset near ∼ 2.2 V (vs. Li/Li^+^), corresponding to the oxidation of S^2‒^ to elemental sulfur (S^0^) [164, 165]. A representative overall oxidation reaction for Li_6_PS_5_Cl is expressed as:

(1) $${\mathrm{Li}}_{6}{\mathrm{PS}}_{5}\mathrm{Cl}\to \frac{1}{2}P_{2}S_{5}+\frac{5}{2}S+\mathrm{LiCl}+5\;{\mathrm{Li}}^{+}+5\;e^{-}$$

The resulting oxidation products possess markedly lower ionic conductivity than the parent SE, leading to increased interfacial resistance and capacity fading. Consequently, engineering the SE itself can be more effective than cathode‐only surface modification, as optimized SE compositions mitigate electrochemical degradation both at the carbon|SE contact and at the CAM|SE interface [112, 166, 167, 168]. As an example, Kim et al. exploited chalcogen–chalcogen interactions between thiol functional groups and sulfide SEs to achieve uniform surface modification [112]. A CEI forming additive containing a trimethylsilyl (TMS) functional group was applied to the LPSCl surface in the form of trimethylsilyl thiol (TMS–SH) (Figure 7f). The TMS‐treated LPSCl paired with bare LCO exhibited superior cycling stability and lower interfacial resistance growth compared with bare LPSCl paired with LiNbO_3_‐coated LCO, highlighting the effectiveness of SE‐centric modification strategies.

### 4.3 Sulfide SE‐Driven Electro‐Chemo‐Mechanical Failure and Healing Strategies

**FIGURE 8:**
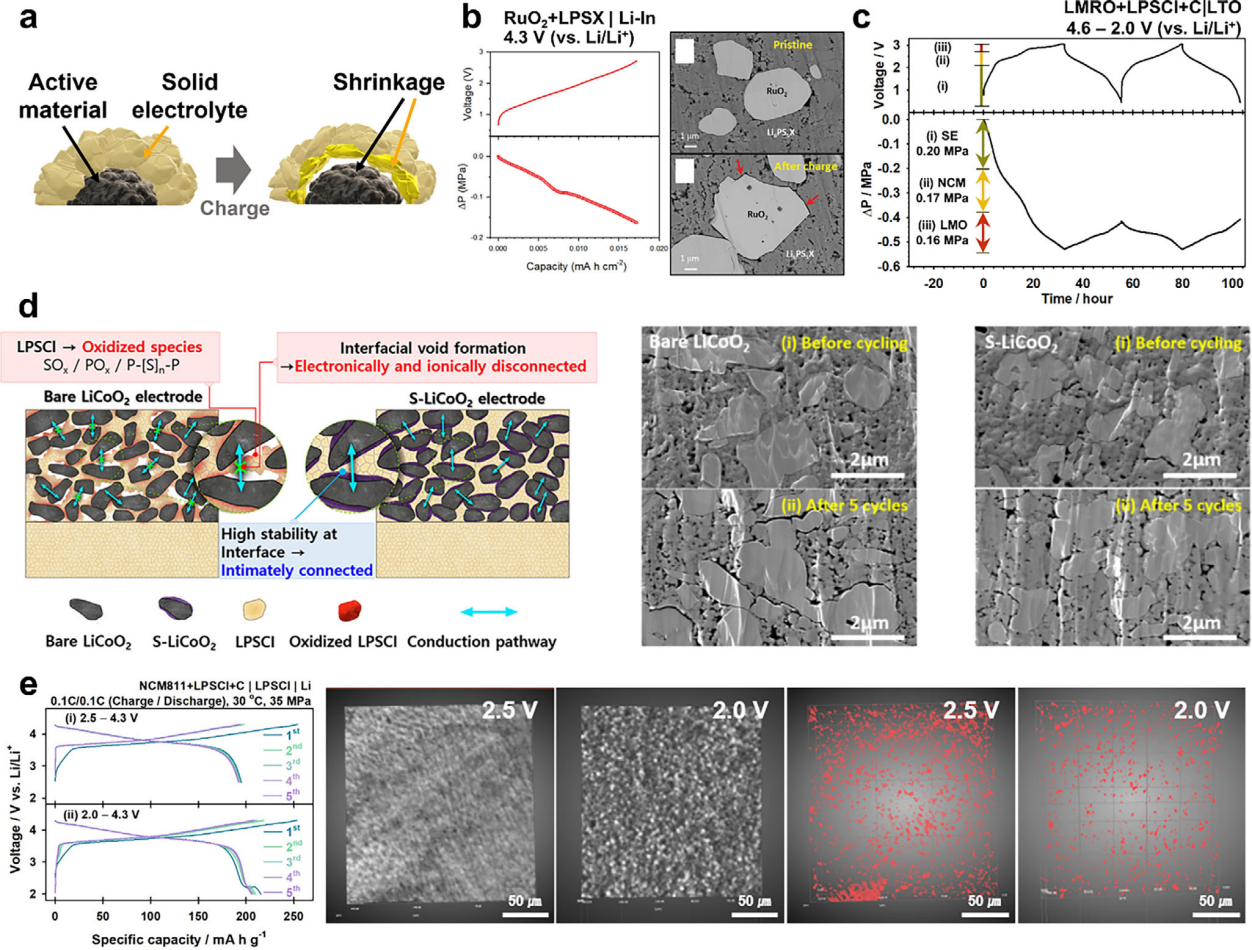
(a) Schematic illustration of SE‐volume‐shrinkage‐induced mechanical failure of ASSBs. Reproduced with permission [113]. Copyright 2025, Wiley‐VCH. (b) In situ DEP results for RuO_2_+LPSCB|LTO cell charged to 4.3 V (vs. Li/Li^+^). Reproduced with permission [84]. Copyright 2021, Wiley‐VCH. (c) In situ DEP result of LMRO+LPSCl+C|LTO cell operated within the voltage range of 3.05 – 0.45 V (vs. LTO), corresponding to 4.6 – 2.0 V(vs. Li/Li^+^). Reproduced with permission [66]. Copyright 2024 Wiley‐VCH. (d) Schematic of interfacial degradation modes (left) and cross sectional images of bare and nano‐structured LCO (right). Reproduced with permission [171]. Copyright 2024, American Chemical Society. (e) Voltage profiles of NCM811|Li in LPSCl‐based ASSBs operated with nominal voltage ranges (2.5 – 4.3 V) and healing voltage ranges (2.0 – 4.3 V) (left), with corresponding 3D rendered X‐ray CT images of the composite cathodes. Reproduced with permission [113]. Copyright 2025, Wiley‐VCH.

Compared to CAMs such as NCM or LFP, sulfide SEs are mechanically more compliant, exhibiting lower Young's modulus and readily densifying under applied stress. Although sulfide SEs were initially expected to buffer CAM dimensional changes, accumulating evidence indicates that they can also actively contribute to electro‐chemo‐mechanical failure. This is due to the volume shrinkage of SEs derived from their oxidative decomposition (Figure 8a) [84, 169, 170]. For example, the delithiation of LPSCl described in Equation (1) reduces its molar volume from 163.7 to 142.8 cm^3^ mol^–^^1^, implying a net particle contraction. Consistent with this, Wang et al. monitored internal pressure evolution in ASSBs using Li_6_PS_5_Cl_0.5_Br_0.5_ (LPSCB) as both the active material and electrolyte, and found that its reaction‐induced volume change between the operating voltages of 0.6 – 3.6 V (vs. Li/Li^+^) exceeded that of Li‐In alloys, $\Delta_{r}{\bar{V}}_{m}$(In/InLi) = +7.89 cm^3^ mol^‒1^ [169]. Moreover, since RuO_2_ is electrochemically inactive but mechanically stable in the typical operating voltage ranges of layered oxide cathode materials, the sole contribution of SE oxidation to the overall cell pressure change during charge was estimated by charging a composite cathode of LPSCB and RuO_2_ mixture to 4.3 V (vs. Li/Li^+^) against zero‐strain Li_4_Ti_5_O_12_. As shown in Figure 8b, the cell pressure decreased monotonically with increasing charge capacity, and cross sectional images revealed interfacial gaps around RuO_2_ particles after charging [84]. In addition, when the LMRO+LPSCl composite cathode was charged, a pronounced pressure drop was observed prior to the delithiation of LMRO during charge (Figure 8c), consistent with sulfide oxidation occurring early and inducing shrinkage of the composite cathode [66]. Such premature SE decomposition not only lowers the effective operating pressure but also accelerates cumulative oxidative degradation and parasitic reactions with CAMs, amplifying the extent of electro‐chemo‐mechanical failure in sulfide‐based ASSBs.

While CAM|SE interfacial detachment has been extensively reported, targeted investigations into the electro‐chemo‐mechanical degradation of the SE itself remain limited. Kim et al. decoupled the effects of CAM volume change and CAM|SE interfacial decomposition by comparing bare and nanostructured LCO cathodes in LPSCl‐based ASSBs, as illustrated in Figure 8d [171]. The nanostructured LCO, whose basal planes were conformally coated with Li_2_SnO_3_ via thermally assisted segregation synthesis, [172] exhibited significantly reduced SE degradation at the CAM surface compared with bare LCO. Because both LCO variants were single‐crystalline and thus experienced similar dimensional changes during cycling, the observed performance enhancement was attributed primarily to improved interfacial stability. Cross sectional SEM and in situ differential electrochemical pressiometry (DEP) confirmed that suppressing CAM|SE interfacial degradation preserved intimate mechanical contact, leading to higher and more stable CAM utilization. These findings demonstrate that the electrochemical stability of the SE interface plays a decisive role in governing the mechanical degradation trajectory of ASSBs.

Enhancing adhesion at the CAM|SE interface represents another effective strategy to mitigate interfacial contact loss. For example, incorporating ductile organic polymers such as polydopamine onto the LPSCl surface has been shown to suppress interfacial degradation, increase particle‐level adhesion, and improve overall electrochemical performance [173]. Lee et al. introduced an electrochemical self‐healing approach by tuning the discharge cut‐off voltage (Figure 8e) [113]. Drawing inspiration from Li‐S redox chemistry, they demonstrated that oxidative decomposition products of LPSCl formed during charging to 4.3 V vs. Li/Li^+^, dominated by oxidation of S^2−^ to S^0^, can be reduced during subsequent discharge. Lowering the discharge cut‐off to 2.0 V versus Li/Li^+^ enabled mechanical healing of the degraded CAM|SE interface, as confirmed by X‐ray CT imaging. This regenerative process enhanced interfacial adhesion and significantly improved cycling stability and rate capability, outperforming LiNbO_3_‐coated NCM cathodes operated with the conventional 2.5 V (vs. Li/Li^+^) discharge cut‐off voltage.

Despite notable progress toward developing SEs with broader electrochemical stability windows, higher ionic conductivity, and improved mechanical compliance, several challenges still remain unsolved for practical implementation. Many reported performance metrics are derived from thin, SE‐rich composite cathodes tested in bulk‐type cells under high stack pressures (often tens of MPa). The following section discusses the mechano‐electrochemical bottlenecks that limit scalable fabrication and reliable operation from the electrode to the cell level and outlines potential design strategies to overcome these challenges.

## 5 Mechano‐Electrochemical Challenges at Electrode‐Cell Levels for Practical Implementation

### 5.1 Fabrication of Practical High Energy Density Electrodes

**FIGURE 9:**
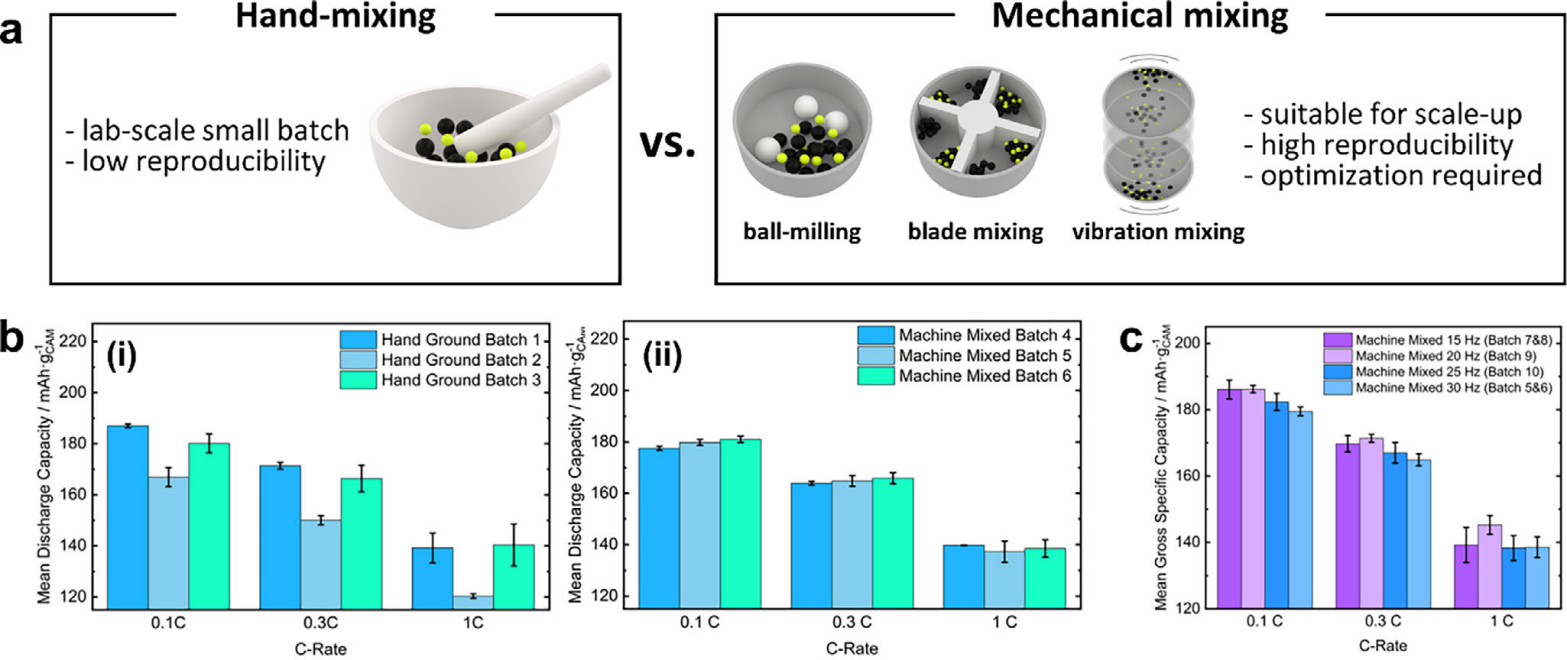
(a) Schematic illustration of hand‐mixing and mechanical‐mixing for fabrication of composite cathode powders for ASSBs. (b) Summarized electrochemical performance of composite cathodes fabricated through (i) hand grinding and (ii) mechanical‐mixing. (c) Changes in specific capacity of composite cathodes mixed through vibrating mill with different mixing frequency. (b,c) Reproduced with permission [178]. Copyright 2025, Wiley‐VCH.

To minimize tortuosity and ensure continuous percolation, homogeneous solid‐solid mixing is essential to distribute the SE as a contiguous network around active material particles. Since both effective ionic conductivity and cell performance are highly sensitive to the degree of mixing, establishing a reproducible and scalable mixing protocol is important. However, most lab‐scale ASSB fabrication still relies on hand‐mixing of electrode powders, a method that suffers from poor reproducibility and is unsuitable for large‐scale production (Figure 9a) [174, 175, 176]. To address this limitation, several scalable mechanical mixing techniques offering tunable shear forces, improved uniformity, and straightforward scale‐up have been developed. Kim et al. reported a blade‐mixing process that delivered superior initial capacity and capacity retention compared with hand‐mixed electrodes, attributed to more homogeneous particle dispersion confirmed by cross sectional SEM [177]. Similarly, Kissel et al. demonstrated that vibrating‐mill mixing produced more reproducible ASSB performance than hand‐mortaring, and that mixing condition should be modulated to achieve best performance (Figure 9b,c) [178]. Liang et al. investigated ball‐milling conditions for NCM811–sulfide SE composite cathodes, showing that while increased rotation speed, milling time, and ball‐to‐powder ratio improved mixing uniformity, excessive processing led to milling‐induced degradation, including Ni reduction in the CAM and sulfur oxidation in the SE [179]. These findings indicate that although mechanical mixing enhances batch‐to‐batch reproducibility, over‐processing can exacerbate parasitic reactions at the CAM|SE interface. This effect is expected to be even more pronounced in polycrystalline cathodes, where particle fracture and enlarged surface area can accelerate interfacial degradation [76, 77, 78, 180]. Overall, optimizing the balance between mixing uniformity and chemical stability remains a key challenge for scalable and reliable ASSB electrode manufacturing.

**FIGURE 10:**
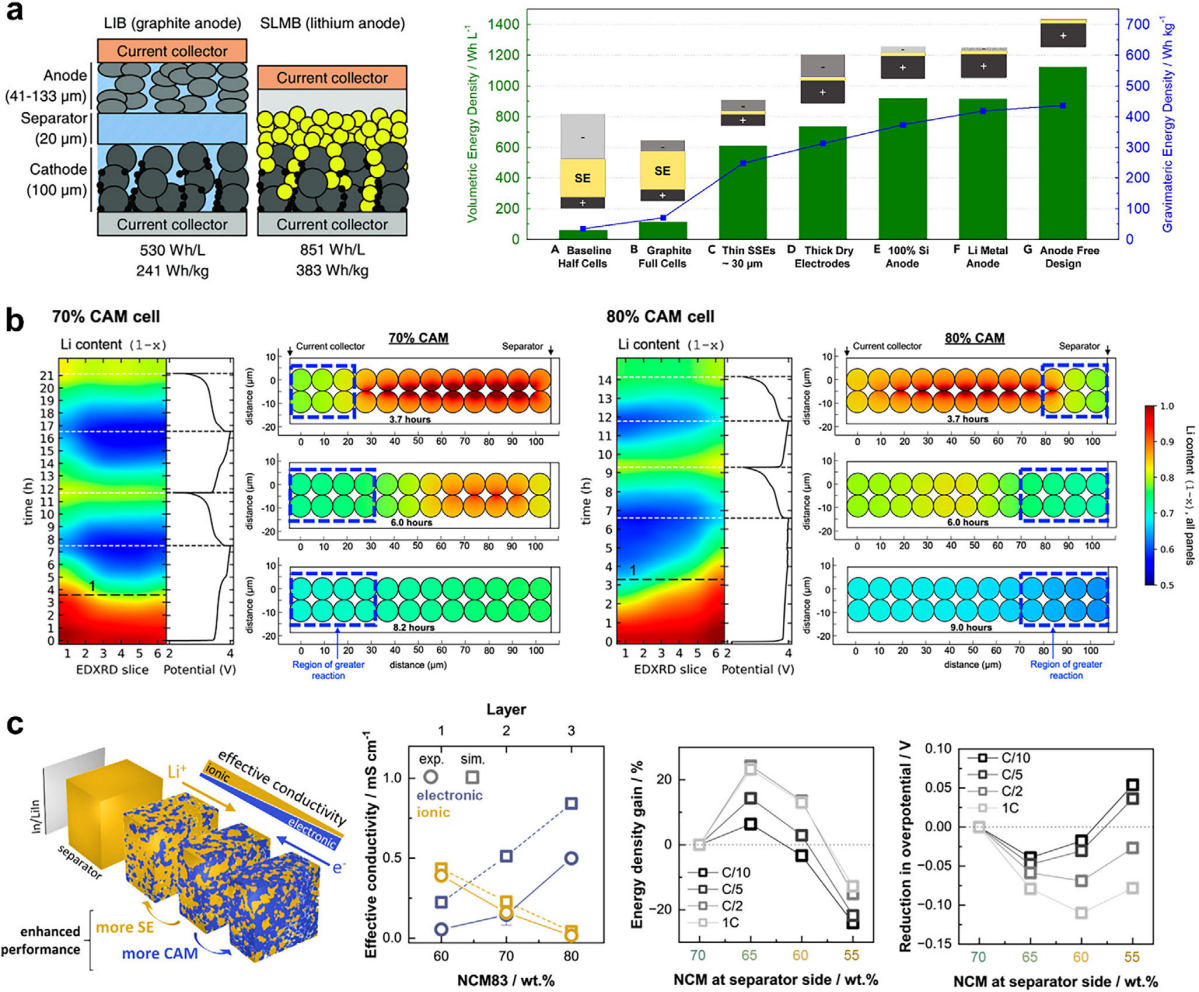
(a) Schematic illustration of LIB and ASSB cell configurations and calculated energy density for ASSBs as a function of cell design parameters. Reproduced with permission [183]. Copyright 2020, Wiley‐VCH. Reproduced with permission [184]. Copyright 2022, Elsevier. (b) Operando XRD results and simulated SOC distribution across the cathode thickness during the first cycle for composite cathodes containing 70% (left) and 80% CAM (right). Reproduced with permission [186]. Copyright 2023, American Chemical Society. (c) Schematic of a conductivity‐graded electrode, measured effective electronic and ionic conductivity plotted against CAM fraction, and the impact of gradient strength on electrochemical performance. Reproduced with permission [188]. Copyright 2025, American Chemical Society.

Once homogeneous solid–solid mixing establishes a continuous percolating network in ASSBs, the operable electrode thickness can exceed that of conventional LIBs [181, 182]. This advantage arises because thick, porous LIB electrodes are inherently limited by liquid‐phase ion transport and electrolyte depletion, whereas ASSB cathodes can form dense solid‐in‐solid composites with built‐in ionic pathways throughout the electrode. To satisfy industrial energy‐density targets of 400 Wh kg^‒1^ or 850 Wh L^‒1^, CAM loadings above 30 mg cm^‒2^ or areal capacities of ca. 5 mA h cm^‒2^ are required (Figure 10a) [13, 183, 184]. However, several studies have shown that gradients in electronic and ionic conductance across the electrode thickness hinder uniform CAM utilization in thick, high‐loading electrodes [185, 186, 187, 188, 189]. Stavola et al. employed spatially resolved operando X‐ray diffraction on composite cathodes comprising 10 µm‐sized polycrystalline NCM111 and LPSCl (Figure 10b) [186]. The observed (003) peak splitting in NCM, which is an indicator of a heterogeneous SOC distribution, varied across the electrode thickness, with the direction of the gradient depending on the CAM/SE ratio. During charging, delithiation near the current collector was favored at low CAM ratios, while the opposite trend was observed at high CAM ratios. The composite containing 70% CAM exhibited the most uniform SOC distribution. As these electrodes were carbon‐free, with NCM serving as the sole electronic conductor, the results underscore that balanced electronic and ionic conductivities are essential for homogeneous utilization in thick ASSB electrodes. To address this, microstructural engineering has been employed to balance charge transport pathways. Shen et al. introduced conductivity‐graded thick cathodes by stacking composite layers with varying carbon contents, constructing a carbon‐rich layer near the current collector and a carbon‐deficient layer near the separator [187]. This configuration yielded superior rate capability and cycling stability compared with non‐graded or reverse‐graded architectures. Schlautmann et al. further elucidated the mechanism through combined experimental and theoretical analyses: ionic pathways near the separator must accommodate the entire Li^+^ flux across the cell, while electronic pathways are primarily active near the current collector (Figure 10c) [188]. Thus, designing electrodes with SE‐rich layers near the separator and CAM‐ or carbon‐rich layers near the current collector minimizes charge‐transport bottlenecks and ensures a more uniform SOC distribution across the electrode thickness. Building upon this concept, Liang and Qian et al. further optimized both carbon content and SE particle size gradients through the electrode depth to fine‐tune electronic and ionic percolation networks [189]. Although conductivity‐graded electrodes have demonstrated clear performance benefits in thick ASSB cathodes, their implementation in mass‐scalable manufacturing remains an ongoing challenge, requiring precise control over layer composition, uniformity, and mechanical integration during fabrication.

**FIGURE 11:**
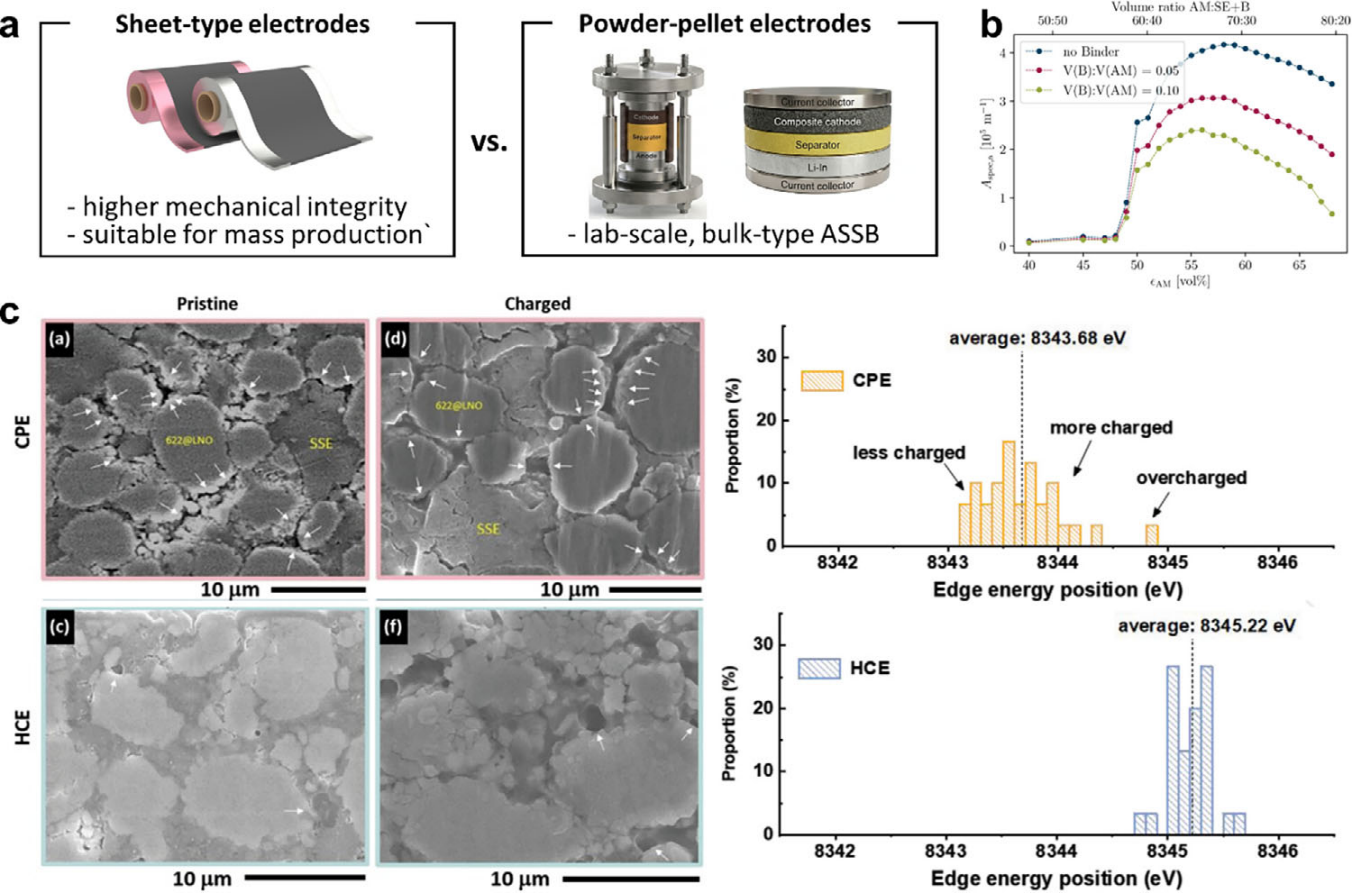
(a) Comparison of sheet‐type and powder‐pellet composite electrodes. (b) Effective conduction surface as a function of CAM/SE ratio and binder content. Reproduced with permission [192]. Copyright 2020, American Chemical Society. (c) Cross sectional SEM images and Ni K‐edge XANES edge energy distributions for cold‐pressed powder‐pellet and hot‐pressed sheet‐type composite cathodes, employing NCM as cathode material. Reproduced with permission [194]. Copyright 2023, Wiley‐VCH.

For mass production of composite cathodes via continuous processes, the use of binder materials is unavoidable. Despite the inherent ductility of sulfide SEs, composite cathode powders are difficult to handle during electrode fabrication [190, 191]. The introduction of a binder, either as a solvent‐based formulation or in a dry form (e.g., polytetrafluoroethylene, PTFE), consolidates discrete particles into cohesive slurries or pastes that can be processed through slurry casting or roll‐to‐roll pressing (Figure 11a). While binders enhance the mechanical integrity of electrodes, they inevitably impede charge‐transport kinetics [192, 193, 194, 195]. Bielefeld et al. modeled percolation behavior in ASSB electrodes with varying binder contents and observed a pronounced decline in the active surface area for both electronic and ionic conduction as the binder fraction increased from 0 to 0.10 [192]. The optimal CAM/SE ratio that maximized interfacial contact area shifted toward lower CAM contents with increasing binder concentration (Figure 11b). Notably, ionic conductivity decreased more sharply than electronic conductivity, as binders preferentially occupy interstitial voids otherwise filled by SEs. Despite these drawbacks, sheet‐type electrodes containing binders have shown improved resistance to mechanical failure during cycling compared with powder‐type electrodes, owing to enhanced interparticle adhesion (Figure 11c) [194]. When compared to cold‐pressed powder electrodes (CPEs), hot‐pressed sheet‐type composite electrodes (HCEs) exhibited reduced particle detachment and higher CAM utilization, as confirmed by Ni K‐edge XANES mapping. Nevertheless, the trade‐off between mechanical robustness and charge‐transport efficiency remains contentious. For instance, Yamamoto et al. demonstrated that binder removal via thermal treatment improved rate capability relative to electrodes containing binders [195].

**FIGURE 12:**
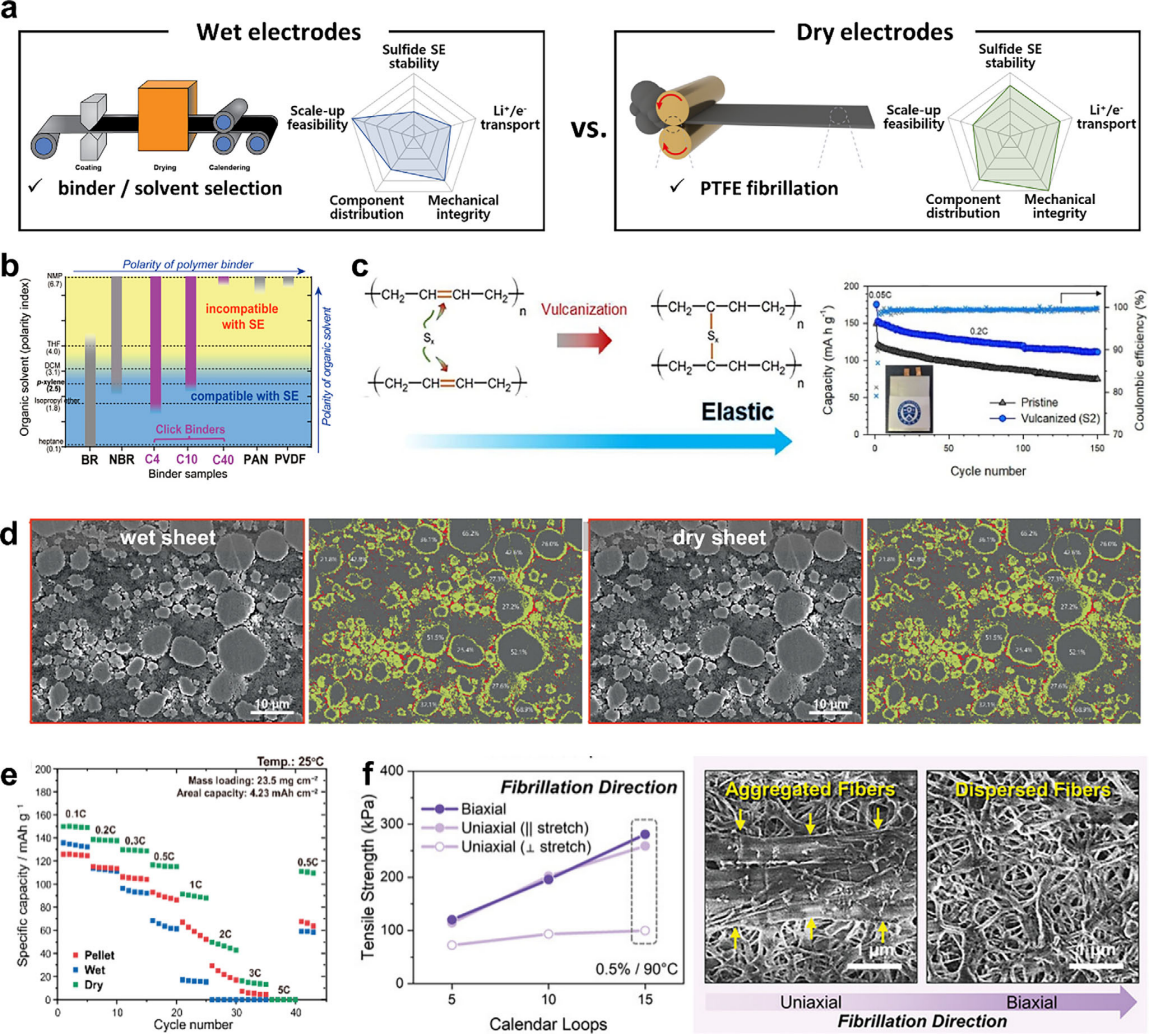
(a) Schematic illustration of wet and dry electrode fabrication methods, and their radar plots in terms of processability and chemical/physical characteristics. Reproduced with permission [196]. Copyright 2024, Elsevier. (b) Binder‐solvent combinations suitable for wet electrode fabrication of sulfide‐based ASSBs. Reproduced with permission [199]. Copyright 2019, American Chemical Society. (c) Schematic of sulfur‐mediated rubber vulcanization and the resulting mechanical/electrochemical performance of sheet‐type ASSBs with and without vulcanization. Reproduced with permission [200]. Copyright 2022, Elsevier. (d) Cross sectional SEM images of wet‐ and dry‐sheet electrodes, where cathode coverage of SE is highlighted in yellow, and (e) comparison of rate performance among powder‐pellet, wet‐sheet and dry‐sheet composite cathodes. Reproduced with permission [212]. Copyright 2024, Springer Nature. (f) Mechanical strength of dry‐sheets as a function of PTFE fibrillation direction. Reproduced with permission [213]. Copyright 2023, Wiley‐VCH.

For sulfide SEs, the key challenge in practical electrode fabrication via slurry casting lies in selecting a chemically compatible binder/solvent system, as sulfides are highly susceptible to oxidative degradation [196, 197, 198, 199]. The trade‐off in performance between wet and dry electrode fabrication methods is summarized by their radar plots in Figure 12a. For wet electrodes, slurry processing is inherently advantageous for scale‐up feasibility, yet it is penalized in sulfide SE stability due to solvent/binder reactivity. Only a limited number of nonpolar or polar aprotic solvents impose minimal damage on sulfide SEs, which consequently restricts viable binder options (Figure 12b). Instead of widely‐used polyvinylidene fluoride (PVdF), rubber‐type binders, such as nitrile butadiene rubber (NBR) and styrene butadiene rubber (SBR), are commonly employed for sheet‐type ASSBs due to their solubility in weakly polar solvents. Considerable efforts have been devoted to enhancing the mechanical robustness of rubber binders through molecular engineering. Kwon et al. introduced a sulfur vulcanization approach by adding elemental sulfur during slurry fabrication (Figure 12c) [200]. The reduction of sulfur opens the C═C double bonds in butadiene rubber (BR), forming inter‐molecular sulfur bridges that reinforce cohesion within the electrode. This strategy enabled NCM711 cathodes to deliver ∼160 mA h g^‒1^ at a relatively low operating pressure of 2 MPa. Similarly, sulfur vulcanization was applied to fabricate a free‐standing LPS separator using ethylene sulfide as a reactive agent [201]. Lee et al. enhanced binder–CAM adhesion by introducing hydrogen‐bonding functionality via a thiol–ene click reaction [199]. The linear C═C double bonds in SBS (polystyrene‐*block*‐polybutadiene‐*block*‐polystyrene) rubber were reduced by the thiol group of mercaptocarboxylic acid, producing hydroxyl groups capable of bonding with surface oxygen atoms on the CAM. Beyond nonpolar rubbers, Jeong et al. showed that using a sulfide‐tolerant ester solvent of octyl acetate enables slurry processing of polyvinylidene fluoride‐chlorotrifluoroethylene (PVdF‐CTFE), whose electronegative F/Cl and polarizable C─F bonds strengthen Li^+^‐mediated electrostatic interactions and binder‐particle adhesion, thereby improving component dispersion and reducing interfacial resistance in composite cathodes [202]. In addition, improving electrode ionic conductivity through solid electrolyte infiltration or lithium salt incorporation during slurry fabrication has also been reported to enhance ASSB performance, demonstrating the importance of integrating mechanical and ionic design principles in binder development [98, 203, 204, 205].

Despite notable advances in binders for wet electrodes, solvent‐driven chemical degradation of sulfide SEs during slurry casting remains an unavoidable drawback of slurry‐casting processes. As an alternative, dry‐sheet fabrication, typically employing PTFE binders, has garnered considerable attention because the direct solvent exposure step of sulfides can be eliminated (Figure 12a) [206, 207, 208, 209, 210, 211]. In addition to eliminating the need for solvents and energy‐intensive drying steps, PTFE‐based dry electrodes require only 0.1–2 wt.% binder yet achieve superior mechanical strength through the unique fibrillation behavior of PTFE under shear force and thermal treatment, which interweaves active material and SE particles into a cohesive matrix [7, 206]. For sulfide‐based ASSBs, this method has enabled cathode areal loadings up to **∼**40 mg cm^−2^. Lee et al. demonstrated that dry electrodes outperform both powder‐pressed and slurry‐cast counterparts, attributing their superior electrochemical performance to reduced particle rearrangement during fabrication (Figure 12d,e) [212]. Digital image analysis of cross sections revealed more uniform SE coverage and higher CAM|SE interfacial connectivity in dry electrodes, whereas solvent evaporation during slurry processing caused binder and carbon migration, leading to heterogeneous particle distribution in wet electrodes. However, from a manufacturing standpoint, scaling up dry‐electrode processing presents challenges. PTFE‐based systems require precise mixing and calendaring control, and their technology readiness level (TRL) remains relatively low, even within LIB manufacturing [207]. Using PTFE as a binder for dry LPSCl separator sheets, Lee et al. investigated how processing parameters affect mechanical integrity (Figure 12f) [213]. The degree of PTFE fibrillation depended strongly on both the number and direction of calendaring passes, with biaxial fibrillation yielding tensile strengths nearly three times higher than uniaxial processing, underscoring the pivotal role of calendaring conditions in determining dry‐sheet quality. PTFE particle size has also been suggested as an important initial condition that determines binder distribution within the sheet, and thereby the electrochemical quality of dry sheets [214]. Beyond improving TRL, another major challenge in dry‐electrode fabrication is preventing disintegration and cracking of cathode particles, particularly in polycrystalline materials, where mechanical stress accumulation during processing can compromise structural integrity and long‐term performance.

### 5.2 Impact of External Pressure during Fabrication and Operation of ASSBs

**FIGURE 13:**
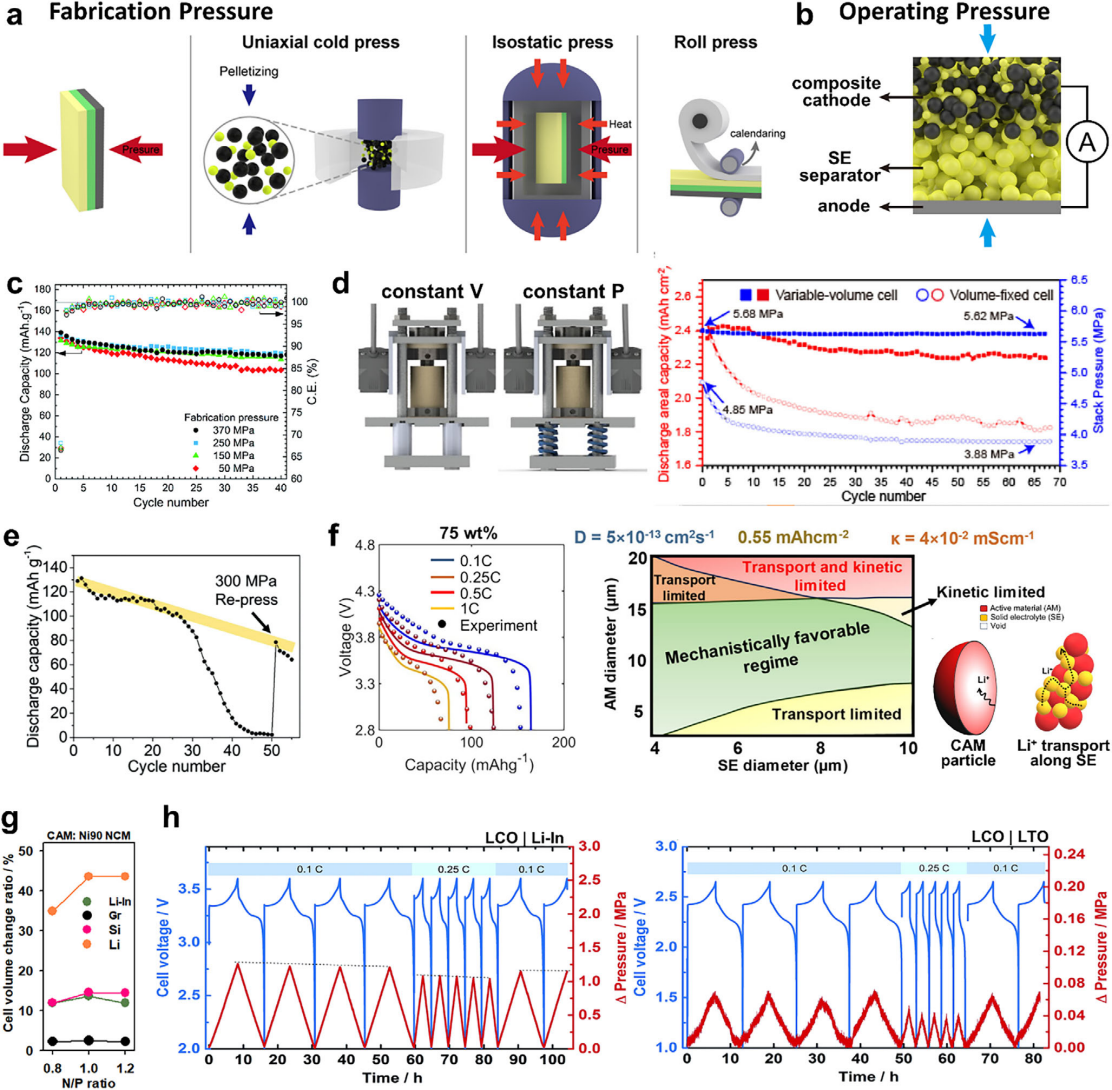
(a) Schematic illustration of fabrication pressure and its application methods. (b) Schematic illustration of operating pressure. (c) Cycle performance of ASSBs as a function of fabrication pressure. Reproduced with permission [217]. Copyright 2020, Royal Society of Chemistry. (d) Electrochemical performance of ASSBs under constant‐volume versus constant‐pressure operation. Reproduced with permission [222]. Copyright 2024, Elsevier. (e) Cycling performance before and after restoring the operating pressure. Reproduced with permission [223]. Copyright 2020, Royal Society of Chemistry. (f) Model‐experiment comparison of ASSB rate performance and mechanistic limitation map plotted against constituent particle sizes with fixed Li^+^ diffusivity, conductivity and cathode loading. Reproduced with permission [226]. Copyright 2025, American Chemical Society. (g) Calculated volume change ratio of a cell comprised of NCM with Ni of 90% as cathode and Li‐In alloy, graphite, silicon or Li metal as anode with varying N/P ratio. (h) In situ DEP results for LCO|Li‐In and LCO|LTO. Reproduced with permission [229]. Copyright 2024, Elsevier.

Although electrochemically active cells can be assembled through simple mechanical mixing and cold pressing in sulfide‐based ASSBs, their cell performance remains highly sensitive to the applied external pressure. The critical role of pressure has been underscored by inter‐laboratory studies using identical SEs and CAMs, which revealed substantial variations not only in SE ionic conductivity but also in initial capacity and capacity retention [174, 215]. These discrepancies highlight the challenges of comparing ASSB performance across different cell architectures and emphasize the need for a mechano‐electrochemical standard for evaluating ASSB behavior. External pressure in ASSBs can be divided into two categories: (i) fabrication pressure, applied during electrode and cell assembly (Figure 13a), and (ii) operating pressure, applied during electrochemical cycling (Figure 13b). Fabrication pressure determines the porosity and particle contact quality within the pristine composite electrode, thereby governing the feasibility of electrochemical operation [216, 217, 218]. Increasing fabrication pressure enhances particle compaction and reduces porosity, leading to improved effective conductance and capacity retention (Figure 13c). However, performance gains saturate beyond a threshold, as packing density cannot exceed the close‐packing limit. Thus, optimized pressurization protocols that achieve intimate interparticle contact at moderate pressures are crucial for practical applications.

While most lab‐scale ASSBs are fabricated by uniaxial hydraulic pressing, isostatic pressing has been shown to produce superior electrode uniformity and electrochemical performance [219]. In simulations of NCM523–LPSCl composite cathodes, Alabdali et al. demonstrated that isostatic pressing imposed less localized stress on CAM particles while achieving comparable compaction and conductivity to uniaxial pressing [220]. From an industrial standpoint, a major challenge lies in developing continuous pressurization processes compatible with mass production. Although high‐performance pouch‐type ASSBs employ isostatic pressing, this method remains batch‐based by nature. Future research should therefore focus on adapting isostatic pressing for continuous manufacturing or on designing electrodes compatible with roll‐to‐roll press fabrication, bridging the gap between laboratory optimization and scalable production.

Modulating the operating pressure of ASSBs can effectively mitigate contact loss arising from particle‐scale dimensional changes during cycling. Applying higher operating pressures suppresses mechanical detachment and enhances CAM utilization in composite electrodes [221, 222]. Two principal operation modes are commonly employed: constant‐volume and constant‐pressure configurations (Figure 13d). In the constant‐volume mode, particle volume changes during cycling lead to fluctuations in internal pressure. Upon initial charging, layered oxides typically contract, while the oxidative decomposition of sulfide SEs further reduces their molar volume [66, 112, 113]. The resulting pressure drop, often detected by in situ pressiometry, indicates that lost interparticle contacts are difficult to recover under constant‐volume conditions. In contrast, constant‐pressure operation incorporates compliant buffer components that accommodate particle volume variations without altering the overall cell pressure. This configuration generally yields better electrochemical performance compared with constant‐volume operation [14, 222]. However, while constant‐pressure operation improves cycling stability, it does so at the expense of pack‐level energy density, as it requires additional mechanical hardware to maintain pressure. Recognizing that CAMs are the primary contributors to pressure loss, Koerver et al. suggested that designing a composite cathode with a mixture of LCO and NCM811 can result in negligible pressure variation during cycling, but with slightly lower energy density than composite cathode comprised of NCM811 only [35].

However, the importance of operating pressure regarding ASSB cycle and rate performance is questionable since approaches that rely primarily on elevating external pressure tend to deliver only modest, short‐lived improvements. For instance, re‐pressurizing already‐operated constant‐volume cells to restore the initial stack pressure results in a temporary increase in discharge capacity but offers negligible improvement in long‐term capacity retention (Figure 13e) [223, 224]. This behavior suggests that nanoscale interfacial detachments and chemically degraded interfaces cannot be fully repaired through mechanical reinforcement alone. Naik et al. further systematically investigated the influence of operating pressure using single‐crystalline NCM523 paired with LPSCl, revealing that at high C‐rates the deliverable capacity is governed primarily by intrinsic limitations, such as tortuous Li^+^ transport pathways within the composite cathode and finite Li^+^ transfer kinetics at the CAM|SE interface, thereby diminishing the marginal benefit of improved solid‐solid contact under elevated pressure [225]. Consistently, Sharma and Vishnugopi et al. suggested that as the current increases, the rate‐determining process can shift from interfacial charge transfer (kinetic‐limited) toward Li^+^ transport through the percolating network of SE particles (transport‐limited) and that resulting mechanistically favorable window emerges only when particle sizes and intrinsic properties of Li^+^ conductivity and diffusivity are co‐optimized (Figure 13f) [226]. Collectively, these works imply that operating pressure is best viewed as a supporting lever that is useful for mitigating cycling‐induced contact loss and suppressing abrupt loss of percolation, but insufficient to overcome overpotential growth rooted in interphase resistance accumulation and charge transport bottlenecks under high current bias.

In this regard, cell architectures that intentionally exploit anode breathing to compensate cathode‐driven pressure decay can be advantageous for full‐cell ASSBs operated under constant‐volume or low‐pressure. Because many practical anodes exhibit much larger absolute volume change than layered oxide cathodes, such as approximately 13% for graphite and over 300% for silicon, the anode expansion can, in principle, provide a built‐in mechanical actuator that partially restores stack pressure as the composite cathode contracts during cycling [227, 228, 229]. Figure 13g illustrates the calculated volume change ratio of a cell comprised of NCM with Ni of 90% as cathode and Li‐In alloy, graphite, silicon or Li metal as anode with varying N/P ratio. The results indicate although the extent depends on N/P ratio, the anode volume change is more significant than cathodes, thereby determining the overall cell volume expansion ratio. This corresponds with in situ pressiometry results of LCO|Li‐In and LCO|LTO cells (Figure 13h), where the measured pressure evolution reflects the dominant role of the electrode with larger mechanical breathing amplitude [229]. Importantly, this mechanical cross‐talk in ASSB provides a new design parameter, since by matching the direction and magnitude of electrode volume changes, the anode can act as an mechanical buffer that mitigates cathode‐induced interparticle contact and aids maintaining ionic percolation at the cathode‐electrolyte interface. However, this utility also depends on the reversibility and kinetics of anode materials. If the anode expansion is highly hysteric or if solid electrolytes and the interphases experience creeping or relaxation prior to the compensation by breathing effect, the operating pressure gain may be marginal. Thus, translating mechanical cross‐talk into a durable strategy requires co‐optimizing of N/P ratio and SOC window, along with assurance that the resulting pressure does not trigger another failure mode, such as electrolyte fracture and dendritic Li metal growth.

In practical applications, operating ASSBs at low external pressure is highly desirable for broader commercial viability. End users typically expect operating pressures of only a few MPa, comparable to LIBs, and anode‐free or anode‐less cell designs, which target higher energy densities, tend to operate more stably at low pressures where dendritic Li growth is effectively suppressed [230, 231]. One approach to improve interfacial contact under such conditions involves adding a small amount of liquid electrolyte to fill interparticle voids and facilitate ionic transport [232]. However, unlike oxide‐based systems, sulfide SEs exhibit poor compatibility with conventional liquid electrolytes, and only a few low‐polarity solvents are suitable. Recently, low‐pressure operation of sheet‐type ASSBs has been demonstrated through optimized fabrication‐pressure protocols. Lee et al. reported a co‐rolling process that simultaneously laminated composite cathodes with separator layers, forming mechanically integrated interfaces suitable for pouch‐type full cells with energy densities approaching ∼800 Wh L^‒1^ [233]. While polycrystalline cathodes tended to fracture under shear stress, single‐crystalline cathodes maintained structural integrity and exhibited excellent cycling durability: electrodes with 5 mA h cm^‒2^ areal capacity retained over 80% of their initial capacity after 500 cycles at an operating pressure of just 2 MPa.

## 6 Summary and Outlook

In this review, we demonstrated the major failure modes of composite cathodes in sulfide‐based ASSBs and introduced various engineering strategies developed to mitigate them, with particular emphasis on the interplay between electrochemical and mechanical processes arising from the absence of electrolyte fluidity. At the materials level, dimensional changes in CAMs are identified as the primary source of electro‐chemo‐mechanical degradation, which can be mitigated through morphological and compositional engineering. Concurrently, compositional and structural tailoring of sulfide SEs not only reinforces their role as fast ionic conductors but also plays a critical part in controlling electro‐chemo‐mechanical failure, particularly through their interfacial degradation on CAM and carbon surfaces.

As growing evidence shows that simply raising operating pressure rarely restores nanoscale conductance once lost, closing the diagnostic gaps should anchor mechanism‐guided ASSB design strategies. In this regard, we further emphasize that the essence of ASSB failure should be interpreted in terms of conductance rather than conductivity, because cell performance depends on both local transport properties and the continuity of percolating pathways. Accordingly, engineering strategies must address both the compositional evolution of interphases, which governs local conductivity, and the mechanical integrity among particles, which dictates the effective charge‐transfer length and pathway continuity. However, disentangling these coupled factors is difficult because the relevant interfaces are barely detectable and distinguishable using conventional characterization techniques. While advanced characterization techniques, such as Raman spectroscopy, XPS, ToF‐SIMS, and TEM provide valuable insight into interfacial chemistry before and after cycling, but often lack the spatial resolution or 3D context to separate CAM|SE and carbon|SE interfaces. On the mechanics side, cross sectional imaging and X‐ray CT reconstructions visualize sub‐micrometer voids and in situ pressiometry tracks cell‐level pressure, yet methods that quantify nanoscale adhesion and local strain within the composite cathodes remain scarce. Thus, in situ characterizations that follow the dynamic evolution of buried interfaces are essential.

While the development of sulfide SEs which allow facile ASSB assembly has significantly enhanced the commercial feasibility of all‐solid‐state battery technology, translating the electrochemical performance demonstrated in lab‐scale bulk‐type cells into practical, high‐energy‐density pouch‐type cells yet remains a major challenge. From the perspective of electrode fabrication, precise optimization of the composition and particle size of each constituent ‒ CAM, SE, conductive carbon, and binder ‒ is critical to achieving a balanced electronic and ionic conduction network. Establishing uniform distribution of the electrode constituents that ensures continuous charge‐transport pathways is also pivotal to attain stable cell performance, especially for thick electrodes. Despite the absence of liquid‐phase mass‐transfer limitations, microstructural engineering remains critical to balance electronic and ionic conductance across the electrode thickness in ASSBs. At the pouch‐cell level, additional engineering strategies are required to preserve percolation pathways during electrochemical cycling, particularly under low‐pressure operating conditions. Ultimately, realizing high‐performance ASSBs at low operating pressures will require integrated efforts spanning materials design, electrode architecture, and cell‐level engineering.

Further considerations for the commercialization of sulfide‐based ASSBs are also suggested as follows:

(1) *Dry‐room compatibility*

For the mass production of sulfide‐based ASSBs, solid electrolytes should be processable under dry‐room conditions, unlike lab‐scale Ar‐filled glove boxes. However, even ppm‐level concentrations of H_2_O and O_2_ have been reported to oxidize thiophosphate building blocks, thereby forming resistive interfaces and reducing overall ionic conductivity [143, 234, 235, 236]. Furthermore, toxic and corrosive H_2_S gas evolution has been reported upon their exposure to trace moisture, which not only corrodes metallic processing equipment but also poses significant environmental, health, and safety (EHS) risks [237]. Encouragingly, several compositional and interfacial engineering strategies have improved the dry‐room compatibility of sulfide solid electrolytes. These include the development of changing halide compositions and introducing dopants to reduce the reactivity of sulfur species, and surface modification with air or moist‐resistant materials, such as oxide or hydrophobic organic molecules [238, 239, 240, 241]. Moreover, moderate post‐exposure annealing has been shown to partially restore performance by removing adsorbed moisture and weakly bound oxidized species [242, 243]. When oxidative degradation has not penetrated deeply into the bulk particle structure, such treatments can yield substantial recovery of ionic conductivity and reduction of interfacial resistance.

(2) *Increasing reports on thermal hazards*

Although sulfide SEs were initially regarded as flame‐retardant, growing evidences show that composite cathodes become thermally unstable when sulfides contact highly delithiated Ni‐rich layered oxides [244, 245, 246, 247, 248, 249]. The ignition temperature of composite cathodes decreased when retrieved at higher SOCs, implicating lattice‐oxygen release as the primary trigger. On the other hand, fully charged LFP‐LPSCl composite cathodes exhibited minimal hazards, consistent with the absence of lattice‐oxygen evolution. Comparisons with halide SEs further indicated that the sulfide SEs also participate in the exothermic pathway, and that severity varies with SE chemistry, particularly regarding their crystallinity and the presence of free sulfur within the crystal structure. Taken together, thermal runaway in sulfide‐based ASSBs is a coupled CAM‐SE phenomenon driven by highly exothermic reactions between oxygen released from the CAM and sulfur‐containing species in the SE. Yet, the quantitative partitioning of heat among (i) lattice‐oxygen loss from the CAM, (ii) oxidation of the sulfide SE, and (iii) CAM surface reconstruction into spinel or rock‐salt phases remains poorly understood, hindering rational mitigation strategies. Along with electro‐chemical origin of thermal instability, mechanical initiators of these incidents should also be assessed [246, 247]. While mechanical friction has been reported to trigger explosive reaction between high‐SOC CAM and sulfide solid electrolyte powder, increasing compaction pressure of composite cathodes led to decreasing heat evolution. In order to fully understand the complicate electro‐chemo‐mechanical coupling regarding thermal instability of composite cathodes, standardized calorimetric and gas‐analysis protocols, conducted under controlled SOC, pressure, and composite‐cathode compositions, is therefore urgently needed. These efforts will deconvolute the interfacial and bulk contributions and guide robust designing rules for thermal stabilization [250, 251].

## Funding

This work was supported by the National Research Foundation of Korea (NRF) (RS‐2024‐00408823 and RS‐2025‐23523434).

## Conflicts of Interest

The authors declare no conflict of interest.

## Data Availability

The authors have nothing to report.

## References

[1] Z. Zhang , Y. Shao , B. Lotsch , et al., “New Horizons for Inorganic Solid State Ion Conductors,” Energy & Environmental Science 11, no. 8 (2018): 1945–1976, 10.1039/C8EE01053F.

[2] T. Placke , R. Kloepsch , S. Dühnen , and M. Winter , “Lithium Ion, Lithium Metal, and Alternative Rechargeable Battery Technologies: The Odyssey for High Energy Density,” Journal of Solid State Electrochemistry 21, no. 7 (2017): 1939–1964, 10.1007/s10008-017-3610-7.

[3] Y. Tian , G. Zeng , A. Rutt , et al., “Promises and Challenges of Next‐Generation “Beyond Li‐Ion” Batteries for Electric Vehicles and Grid Decarbonization,” Chemical Reviews 121, no. 3 (2021): 1623–1669. 33356176 10.1021/acs.chemrev.0c00767

[4] A. Manthiram , X. Yu , and S. Wang , “Lithium Battery Chemistries Enabled by Solid‐State Electrolytes,” Nature Reviews Materials 2, no. 4 (2017): 16103.

[5] Y. S. Meng , V. Srinivasan , and K. Xu , “Designing Better Electrolytes,” Science 378, no. 6624: abq3750, 10.1126/science.abq3750. 36480630

[6] P. Albertus , V. Anandan , C. Ban , et al., “Challenges for and Pathways toward Li‐Metal‐Based All‐Solid‐State Batteries,” ACS Energy Letters 6, no. 4 (2021): 1399–1404.

[7] Z. Zhang , L. Wu , D. Zhou , W. Weng , and X. Yao , “Flexible Sulfide Electrolyte Thin Membrane with Ultrahigh Ionic Conductivity for All‐Solid‐State Lithium Batteries,” Nano Letters 21, no. 12 (2021): 5233–5239. 34106717 10.1021/acs.nanolett.1c01344

[8] K. J. Kim , M. Balaish , M. Wadaguchi , L. Kong , and J. L. M. Rupp , “Solid‐State Li–Metal Batteries: Challenges and Horizons of Oxide and Sulfide Solid Electrolytes and Their Interfaces,” Advanced Energy Materials 11, no. 1 (2021): 2002689.

[9] Y. Wu , S. Wang , H. Li , L. Chen , and F. Wu , “Progress in Thermal Stability of All‐Solid‐State‐Li‐Ion‐Batteries,” InfoMat 3, no. 8 (2021): 827–853.

[10] Z. Deng , Z. Wang , I.‐H. Chu , J. Luo , and S. P. Ong , “Elastic Properties of Alkali Superionic Conductor Electrolytes from First Principles Calculations,” Journal of The Electrochemical Society 163, no. 2 (2016): A67–A74, 10.1149/2.0061602jes.

[11] A. Kato , M. Yamamoto , A. Sakuda , A. Hayashi , and M. Tatsumisago , “Mechanical Properties of Li_2_S–P_2_S_5_ Glasses with Lithium Halides and Application in All‐Solid‐State Batteries,” ACS Applied Energy Materials 1, no. 3 (2018): 1002–1007, 10.1021/acsaem.7b00140.

[12] Y.‐G. Lee , S. Fujiki , C. Jung , et al., “High‐Energy Long‐Cycling All‐Solid‐State Lithium Metal Batteries Enabled by Silver–Carbon Composite Anodes,” Nature Energy 5, no. 4 (2020): 299–308, 10.1038/s41560-020-0575-z.

[13] S. Randau , D. A. Weber , O. Kötz , et al., “Benchmarking the Performance of All‐Solid‐State Lithium Batteries,” Nature Energy 5, no. 3 (2020): 259–270, 10.1038/s41560-020-0565-1.

[14] D. H. S. Tan , Y.‐T. Chen , H. Yang , et al., “Carbon‐Free High‐Loading Silicon Anodes Enabled by Sulfide Solid Electrolytes,” Science 373, no. 6562 (2021): 1494–1499. 34554780 10.1126/science.abg7217

[15] S. Kalnaus , N. J. Dudney , A. S. Westover , E. Herbert , and S. Hackney , “Solid‐State Batteries: The Critical Role of Mechanics,” Science 381, no. 6664: abg5998, 10.1126/science.abg5998. 37733866

[16] G. F. Dewald , S. Ohno , M. A. Kraft , et al., “Experimental Assessment of the Practical Oxidative Stability of Lithium Thiophosphate Solid Electrolytes,” Chemistry of Materials 31, no. 20 (2019): 8328–8337, 10.1021/acs.chemmater.9b01550.

[17] T. Hakari , M. Deguchi , K. Mitsuhara , et al., “Structural and Electronic‐State Changes of a Sulfide Solid Electrolyte during the Li Deinsertion–Insertion Processes,” Chemistry of Materials 29, no. 11 (2017): 4768–4774.

[18] A. J. Louli , J. Li , S. Trussler , C. R. Fell , and J. R. Dahn , “Volume, Pressure and Thickness Evolution of Li‐Ion Pouch Cells with Silicon‐Composite Negative Electrodes,” Journal of The Electrochemical Society 164, no. 12 (2017): A2689–A2696, 10.1149/2.1691712jes.

[19] P. Lennartz , B. A. Paren , A. Herzog‐Arbeitman , et al., “Practical Considerations for Enabling Li|Polymer Electrolyte Batteries,” Joule 7, no. 7 (2023): 1471–1495.

[20] S. Watanabe , M. Kinoshita , T. Hosokawa , K. Morigaki , and K. Nakura , “Capacity Fade of LiAl_y_Ni_1−x−y_Co_x_O_2_ Cathode for Lithium‐Ion Batteries during Accelerated Calendar and Cycle Life Tests (Surface Analysis of LiAl_y_Ni_1−x−y_Co_x_O_2_ Cathode After Cycle Tests in Restricted Depth of Discharge Ranges),” Journal of Power Sources 258 (2014): 210–217, 10.1016/j.jpowsour.2014.02.018.

[21] N.‐Y. Park , G.‐T. Park , S.‐B. Kim , W. Jung , B.‐C. Park , and Y.‐K. Sun , “Degradation Mechanism of Ni‐Rich Cathode Materials: Focusing on Particle Interior,” ACS Energy Letters 7, no. 7 (2022): 2362–2369.

[22] B. L. D. Rinkel , D. S. Hall , I. Temprano , and C. P. Grey , “Electrolyte Oxidation Pathways in Lithium‐Ion Batteries,” Journal of the American Chemical Society 142, no. 35 (2020): 15058–15074. 32697590 10.1021/jacs.0c06363

[23] W. M. Dose , W. Li , I. Temprano , et al., “Onset Potential for Electrolyte Oxidation and Ni‐Rich Cathode Degradation in Lithium‐Ion Batteries,” ACS Energy Letters 7, no. 10 (2022): 3524–3530. 36277132 10.1021/acsenergylett.2c01722 PMC9578037

[24] Y. Zhang , Y. Katayama , R. Tatara , et al., “Revealing Electrolyte Oxidation via Carbonate Dehydrogenation on Ni‐Based Oxides in Li‐Ion Batteries by In Situ Fourier Transform Infrared Spectroscopy,” Energy & Environmental Science 13, no. 1 (2020): 183–199, 10.1039/C9EE02543J.

[25] H. Kwon , H. Kim , J. Hwang , et al., “Borate–Pyran Lean Electrolyte‐Based Li‐Metal Batteries with Minimal Li Corrosion,” Nature Energy 9, no. 1 (2024): 57–69.

[26] D.‐S. Ko , J.‐H. Park , S. Park , et al., “Microstructural Visualization of Compositional Changes Induced by Transition Metal Dissolution in Ni‐Rich Layered Cathode Materials by High‐Resolution Particle Analysis,” Nano Energy 56 (2019): 434–442, 10.1016/j.nanoen.2018.11.046.

[27] Y. Song , L. Wang , L. Sheng , et al., “The Significance of Mitigating Crosstalk in Lithium‐Ion Batteries: A Review,” Energy & Environmental Science 16, no. 5 (2023): 1943–1963.

[28] D. R. Gallus , R. Schmitz , R. Wagner , et al., “The Influence of Different Conducting Salts on the Metal Dissolution and Capacity Fading of NCM Cathode Material,” Electrochimica Acta 134 (2014): 393–398, 10.1016/j.electacta.2014.04.091.

[29] J. Hong , H.‐D. Lim , M. Lee , et al., “Critical Role of Oxygen Evolved from Layered Li–Excess Metal Oxides in Lithium Rechargeable Batteries,” Chemistry of Materials 24, no. 14 (2012): 2692–2697, 10.1021/cm3005634.

[30] H. Liu , M. Wolfman , K. Karki , et al., “Intergranular Cracking as a Major Cause of Long‐Term Capacity Fading of Layered Cathodes,” Nano Letters 17, no. 6 (2017): 3452–3457, 10.1021/acs.nanolett.7b00379. 28548836

[31] G. T. Park , S. B. Kim , B. Namkoong , et al., “Intergranular Shielding for Ultrafine‐Grained Mo‐Doped Ni‐Rich Li[Ni_0.96_Co_0.04_]O_2_ Cathode for Li‐Ion Batteries with High Energy Density and Long Life,” Angewandte Chemie (International Ed in English) 62, no. 52 (2023): 202314480. 10.1002/anie.202314480 37955417

[32] R. Ruess , S. Schweidler , H. Hemmelmann , et al., “Influence of NCM Particle Cracking on Kinetics of Lithium‐Ion Batteries with Liquid or Solid Electrolyte,” Journal of The Electrochemical Society 167, no. 10 (2020): 100532, 10.1149/1945-7111/ab9a2c.

[33] R. Koerver , W. Zhang , L. de Biasi , et al., “Chemo‐Mechanical Expansion of Lithium Electrode Materials—On the Route to Mechanically Optimized All‐Solid‐State Batteries,” Energy & Environmental Science 11, no. 8 (2018): 2142–2158, 10.1039/C8EE00907D.

[34] Y. B. Song , H. Kwak , W. Cho , K. S. Kim , Y. Seok Jung , and K.‐H. Park , “Electrochemo‐Mechanical Effects as a Critical Design Factor for All‐Solid‐State Batteries,” Current Opinion in Solid State and Materials Science 26, no. 1 (2022): 100977.

[35] H. Tsukasaki , M. Otoyama , Y. Mori , et al., “Analysis of Structural and Thermal Stability in the Positive Electrode for Sulfide‐Based All‐Solid‐State Lithium Batteries,” Journal of Power Sources 367 (2017): 42–48, 10.1016/j.jpowsour.2017.09.031.

[36] H. Tsukasaki , T. Uchiyama , K. Yamamoto , et al., “Exothermal Mechanisms in the Charged LiNi_1/3_Mn_1/3_Co_1/3_O_2_ Electrode Layers for Sulfide‐Based All‐Solid‐State Lithium Batteries,” Journal of Power Sources 434 (2019): 226714, 10.1016/j.jpowsour.2019.226714.

[37] A. Kato , M. Nose , M. Yamamoto , A. Sakuda , A. Hayashi , and M. Tatsumisago , “Mechanical Properties of Sulfide Glasses in All‐Solid‐State Batteries,” Journal of the Ceramic Society of Japan 126, no. 9 (2018): 719–727, 10.2109/jcersj2.18022.

[38] S. Choi , M. Jeon , J. Ahn , et al., “Quantitative Analysis of Microstructures and Reaction Interfaces on Composite Cathodes in All‐Solid‐State Batteries Using a Three‐Dimensional Reconstruction Technique,” ACS Applied Materials & Interfaces 10, no. 28 (2018): 23740–23747, 10.1021/acsami.8b04204. 29985582

[39] F. Strauss , T. Bartsch , L. de Biasi , et al., “Impact of Cathode Material Particle Size on the Capacity of Bulk‐Type All‐Solid‐State Batteries,” ACS Energy Letters 3, no. 4 (2018): 992–996, 10.1021/acsenergylett.8b00275.

[40] A. Sakuda , T. Takeuchi , and H. Kobayashi , “Electrode Morphology in All‐Solid‐State Lithium Secondary Batteries Consisting of LiNi_1/3_Co_1/3_Mn_1/3_O_2_ and Li_2_S‐P_2_S_5_ Solid Electrolytes,” Solid State Ionics 285 (2016): 112–117, 10.1016/j.ssi.2015.09.010.

[41] J. Kim , W. Lee , J. Seok , et al., “Inhomogeneous Lithium‐Storage Reaction Triggering the Inefficiency of All‐Solid‐State Batteries,” Journal of Energy Chemistry 66 (2022): 226–236, 10.1016/j.jechem.2021.08.017.

[42] S. Deng , Y. Wang , T. Sun , et al., “Impacts of the Conductive Networks on Solid‐State Battery Operation,” Angewandte Chemie International Edition 64 (2025): 202511534, 10.1002/anie.202511534. PMC12455428 40761037

[43] X. Liu , Y. Cheng , Y. Su , et al., “Revealing the Surface‐to‐Bulk Degradation Mechanism of Nickel‐Rich Cathode in Sulfide All‐Solid‐State Batteries,” Energy Storage Materials 54 (2023): 713–723, 10.1016/j.ensm.2022.11.019.

[44] W. Zhang , D. A. Weber , H. Weigand , et al., “Interfacial Processes and Influence of Composite Cathode Microstructure Controlling the Performance of All‐Solid‐State Lithium Batteries,” ACS Applied Materials & Interfaces 9, no. 21 (2017): 17835–17845. 28481084 10.1021/acsami.7b01137

[45] A. Bielefeld , D. A. Weber , and J. Janek , “Microstructural Modeling of Composite Cathodes for All‐Solid‐State Batteries,” Journal of Physical Chemistry C 123, no. 3 (2019): 1626–1634, 10.1021/acs.jpcc.8b11043.

[46] T. Shi , Q. Tu , Y. Tian , et al., “High Active Material Loading in All‐Solid‐State Battery Electrode via Particle Size Optimization,” Advanced Energy Materials 10, no. 1 (2020): 1902881, 10.1002/aenm.201902881.

[47] A. Bielefeld , D. A. Weber , R. Rueß , V. Glavas , and J. Janek , “Influence of Lithium Ion Kinetics, Particle Morphology and Voids on the Electrochemical Performance of Composite Cathodes for All‐Solid‐State Batteries,” Journal of the Electrochemical Society 169, no. 2 (2022): 020539.

[48] L. de Biasi , A. O. Kondrakov , H. Geßwein , T. Brezesinski , P. Hartmann , and J. Janek , “Between Scylla and Charybdis: Balancing among Structural Stability and Energy Density of Layered NCM Cathode Materials for Advanced Lithium‐Ion Batteries,” Journal of Physical Chemistry C 121, no. 47 (2017): 26163–26171, 10.1021/acs.jpcc.7b06363.

[49] H.‐H. Ryu , B. Namkoong , J.‐H. Kim , I. Belharouak , C. S. Yoon , and Y.‐K. Sun , “Capacity Fading Mechanisms in Ni‐Rich Single‐Crystal NCM Cathodes,” ACS Energy Letters 6, no. 8 (2021): 2726.

[50] R. Jung , M. Metzger , F. Maglia , C. Stinner , and H. A. Gasteiger , “Oxygen Release and Its Effect on the Cycling Stability of LiNi_x_Mn_y_Co_z_O_2_ (NMC) Cathode Materials for Li‐Ion Batteries,” Journal of The Electrochemical Society 164, no. 7 (2017): A1361–A1377, 10.1149/2.0021707jes.

[51] J.‐H. Kim , K.‐J. Park , S. J. Kim , C. S. Yoon , and Y.‐K. Sun , “A Method of Increasing the Energy Density of Layered Ni‐Rich Li[Ni_1−2_ * _x_ *Co* _x_ *Mn* _x_ *]O_2_ Cathodes (x = 0.05, 0.1, 0.2),” Journal of Materials Chemistry A 7, no. 6 (2019): 2694–2701, 10.1039/C8TA10438G.

[52] S. Lee , G. Song , B. Yun , et al., “Revealing the Nanoscopic Corrosive Degradation Mechanism of Nickel‐Rich Layered Oxide Cathodes at Low State‐of‐Charge Levels: Corrosion Cracking and Pitting,” ACS Nano 18, no. 15 (2024): 10566–10581. 38556986 10.1021/acsnano.4c00202

[53] H.‐H. Ryu , K.‐J. Park , C. S. Yoon , and Y.‐K. Sun , “Capacity Fading of Ni‐Rich Li[Ni* _x_ *Co* _y_ *Mn_1–_ * _x_ *—* _y_ *]O_2_ (0.6 ≤ x ≤ 0.95) Cathodes for High‐Energy‐Density Lithium‐Ion Batteries: Bulk or Surface Degradation?,” Chemistry of Materials 30, no. 3 (2018): 1155–1163, 10.1021/acs.chemmater.7b05269.

[54] T. Jousseaume , J.‐F. Colin , M. Chandesris , S. Lyonnard , and S. Tardif , “Strain and Collapse during Lithiation of Layered Transition Metal Oxides: A Unified Picture,” Energy & Environmental Science 17, no. 8 (2024): 2753–2764.

[55] T.‐Y. Yu , H.‐U. Lee , J. W. Lee , et al., “Limitation of Ni‐Rich Layered Cathodes in All‐Solid‐State Lithium Batteries,” Journal of Materials Chemistry A 11, no. 45 (2023): 24629–24636, 10.1039/D3TA05060B.

[56] K.‐J. Park , J.‐Y. Hwang , H.‐H. Ryu , et al., “Degradation Mechanism of Ni‐Enriched NCA Cathode for Lithium Batteries: Are Microcracks Really Critical?,” ACS Energy Letters 4, no. 6 (2019): 1394–1400, 10.1021/acsenergylett.9b00733.

[57] C. S. Yoon , D.‐W. Jun , S.‐T. Myung , and Y.‐K. Sun , “Structural Stability of LiNiO_2_ Cycled above 4.2 V,” ACS Energy Letters 2, no. 5 (2017): 1150–1155.

[58] R. Koerver , F. Walther , I. Aygün , et al., “Redox‐Active Cathode Interphases in Solid‐State Batteries,” Journal of Materials Chemistry A 5, no. 43 (2017): 22750–22760, 10.1039/C7TA07641J.

[59] X. Liu , B. Zheng , J. Zhao , et al., “Electrochemo‐Mechanical Effects on Structural Integrity of Ni‐Rich Cathodes with Different Microstructures in all Solid‐State Batteries,” Advanced Energy Materials 11, no. 8 (2021): 2003583.

[60] F. Strauss , L. de Biasi , A. Y. Kim , et al., “Rational Design of Quasi‐Zero‐Strain NCM Cathode Materials for Minimizing Volume Change Effects in All‐Solid‐State Batteries,” ACS Materials Letters 2, no. 1 (2020): 84–88.

[61] D. Goonetilleke , F. Riewald , A. O. Kondrakov , J. Janek , T. Brezesinski , and M. Bianchini , “Alleviating Anisotropic Volume Variation at Comparable Li Utilization during Cycling of Ni‐Rich, Co‐Free Layered Oxide Cathode Materials,” Journal of Physical Chemistry C 126, no. 40 (2022): 16952–16964.

[62] W. Lee , M. Choi , M. Kim , et al., “Pinning Effects of Heavy Elements for the Structural Stability of Ni‐Based Layered Oxides,” ACS Energy Letters 10, no. 9 (2025): 4527.

[63] H. Kim , A. Choi , S. W. Doo , J. Lim , Y. Kim , and K. T. Lee , “Role of Na^+^ in the Cation Disorder of [Li_1‐x_Na_x_]NiO_2_ as a Cathode for Lithium‐Ion Batteries,” Journal of the Electrochemical Society 165, no. 2 (2018): A201–A205, 10.1149/2.0771802jes.

[64] K. Min , S.‐W. Seo , Y. Y. Song , H. S. Lee , and E. Cho , “A First‐Principles Study of the Preventive Effects of Al and Mg Doping on the Degradation in LiNi_0.8_Co_0.1_Mn_0.1_O_2_ Cathode Materials,” Physical Chemistry Chemical Physics 19, no. 3 (2017): 1762–1769, 10.1039/C6CP06270A. 27886291

[65] T. He , L. Chen , Y. Su , et al., “The Effects of Alkali Metal Ions with Different Ionic Radii Substituting in Li Sites on the Electrochemical Properties of Ni‐Rich Cathode Materials,” Journal of Power Sources 441 (2019): 227195, 10.1016/j.jpowsour.2019.227195.

[66] G. Song , S. Lee , T. Kim , et al., “Mechano‐Electrochemical Behavior of Nanostructured Li‐ and Mn‐Rich Layered Oxides with Superior Capacity Retention and Voltage Decay for Sulfide‐Based All‐Solid‐State Batteries,” Advanced Energy Materials 14, no. 47 (2024): 2403374.

[67] Y. Wu , C. Li , X. Zheng , et al., “High Energy Sulfide‐Based All‐Solid‐State Lithium Batteries Enabled by Single‐Crystal Li‐Rich Cathodes,” ACS Energy Letters 9 (2024): 5156–5165, 10.1021/acsenergylett.4c01764.

[68] S. H. Choi , G. Song , K. Park , et al., “Active–Inactive Molten Salt Synthesis of Li‐ and Mn‐Rich Layered Oxide Single Crystals as Cathode Materials for All‐Solid‐State Batteries,” Chemistry of Materials 36, no. 19 (2024): 9666–9676.

[69] S. H. Jung , U.‐H. Kim , J.‐H. Kim , et al., “Ni‐Rich Layered Cathode Materials with Electrochemo‐Mechanically Compliant Microstructures for All‐Solid‐State Li Batteries,” Advanced Energy Materials 10, no. 6 (2020): 1903360, 10.1002/aenm.201903360.

[70] N.‐Y. Park , H.‐U. Lee , T.‐Y. Yu , et al., “High‐Energy, Long‐Life Ni‐Rich Cathode Materials with Columnar Structures for All‐Solid‐State Batteries,” Nature Energy 10 (2025): 479–489.

[71] T.‐Y. Yu , N.‐Y. Park , H. Kim , et al., “Tuning the Lithium Diffusion Kinetics in Co‐Free Layered Cathodes for High‐Performance All‐Solid‐State Batteries,” ACS Energy Letters 10, no. 5 (2025): 2477–2486.

[72] X. Xu , H. Huo , J. Jian , et al., “Radially Oriented Single‐Crystal Primary Nanosheets Enable Ultrahigh Rate and Cycling Properties of LiNi_0.8_Co_0.1_Mn_0.1_O_2_ Cathode Material for Lithium‐Ion Batteries,” Advanced Energy Materials 9, no. 15 (2019): 1803963, 10.1002/aenm.201803963.

[73] U.‐H. Kim , H.‐H. Ryu , J.‐H. Kim , et al., “Microstructure‐Controlled Ni‐Rich Cathode Material by Microscale Compositional Partition for Next‐Generation Electric Vehicles,” Advanced Energy Materials 9, no. 15 (2019): 1803902, 10.1002/aenm.201803902.

[74] S. Payandeh , C. Njel , A. Mazilkin , et al., “The Effect of Single versus Polycrystalline Cathode Particles on All‐Solid‐State Battery Performance,” Advanced Materials Interfaces 10, no. 3 (2023): 2201806.

[75] X. Li , W. Peng , R. Tian , et al., “Excellent Performance Single‐Crystal NCM Cathode under High Mass Loading for All‐Solid‐State Lithium Batteries,” Electrochimica Acta 363 (2020): 137185, 10.1016/j.electacta.2020.137185.

[76] H. C. W. Parks , A. M. Boyce , A. Wade , et al., “Direct Observations of Electrochemically Induced Intergranular Cracking in Polycrystalline NMC811 Particles,” Journal of Materials Chemistry A 11, no. 39 (2023): 21322–21332, 10.1039/D3TA03057A.

[77] A. Omirkhan , O. Gavalda‐Diaz , S. Wang , I. E. L. Stephens , F. Giuliani , and M. P. Ryan , “Investigating the Effect of Lithiation on Polycrystalline NMC811 Li‐Ion Battery Cathode Cracking Using In Situ SEM Micromechanical Testing,” Energy & Environmental Science 18, no. 20 (2025): 9254–9262.

[78] E. Trevisanello , R. Ruess , G. Conforto , F. H. Richter , and J. Janek , “Polycrystalline and Single Crystalline NCM Cathode Materials—Quantifying Particle Cracking, Active Surface Area, and Lithium Diffusion,” Advanced Energy Materials 11, no. 18 (2021): 2003400.

[79] S.‐B. Hong , Y.‐J. Lee , H.‐J. Lee , et al., “Exploring the Cathode Active Materials for Sulfide‐Based All‐Solid‐State Lithium Batteries with High Energy Density,” Small 20 (2024): 2304747. 10.1002/smll.202304747 37847909

[80] J. Li , A. R. Cameron , H. Li , et al., “Comparison of Single Crystal and Polycrystalline LiNi_0.5_Mn_0.3_Co_0.2_O_2_ Positive Electrode Materials for High Voltage Li‐Ion Cells,” Journal of The Electrochemical Society 164, no. 7 (2017): A1534–A1544, 10.1149/2.0991707jes.

[81] H.‐H. Ryu , S.‐B. Lee , C. S. Yoon , and Y.‐K. Sun , “Morphology‐Dependent Battery Performance of Ni‐Rich Layered Cathodes: Single‐Crystal versus Refined Polycrystal,” ACS Energy Letters 7, no. 9 (2022): 3072–3079.

[82] C. Wang , R. Yu , S. Hwang , et al., “Single Crystal Cathodes Enabling High‐Performance All‐Solid‐State Lithium‐Ion Batteries,” Energy Storage Materials 30 (2020): 98–103, 10.1016/j.ensm.2020.05.007.

[83] J. Y. Jung , K. Kim , J. H. Suh , et al., “Customizing the Morphology and Microstructure of Single‐Crystalline Ni‐Rich Layered Cathode Materials for All‐Solid‐State Batteries,” Chemical Engineering Journal 470 (2023): 144381, 10.1016/j.cej.2023.144381.

[84] Y. Han , S. H. Jung , H. Kwak , et al., “Single‐ or Poly‐Crystalline Ni‐Rich Layered Cathode, Sulfide or Halide Solid Electrolyte: Which Will be the Winners for All‐Solid‐State Batteries?,” Advanced Energy Materials 11 (2021): 2100126.

[85] H. Kim , Y. Kong , W. M. Seong , and A. Manthiram , “Controlling the Microstructure of Cobalt‐Free, High‐Nickel Cathode Materials with Dopant Solubility for Lithium‐Ion Batteries,” ACS Applied Materials Interfaces 15, no. 22 (2023): 26585–26592. 37222422 10.1021/acsami.3c02009

[86] B. Zahiri , A. Patra , C. Kiggins , et al., “Revealing the Role of the Cathode–Electrolyte Interface on Solid‐State Batteries,” Nature Materials 20, no. 10 (2021): 1392–1400. 34017118 10.1038/s41563-021-01016-0

[87] H. Jeon , D.‐H. Kwon , H. Kim , et al., “Tailoring Shape and Exposed Crystal Facet of Single‐Crystal Layered‐Oxide Cathode Particles for All‐Solid‐State Batteries,” Chemical Engineering Journal 445 (2022): 136828, 10.1016/j.cej.2022.136828.

[88] S. Zhang , F. Zhao , L. Li , and X. Sun , “Solid‐State Electrolytes Expediting Interface‐Compatible Dual‐Conductive Cathodes for All‐Solid‐State Batteries,” Energy & Environmental Science 18, no. 13 (2025): 6530–6539.

[89] X. Xiong , X. Ma , T. Lv , L. Chen , and L. Suo , “Monophase‐Homointerface Electrodes Intrinsically Stabilize High‐Voltage All‐Solid‐State Batteries,” Science China Chemistry 67, no. 5 (2024): 1729–1739.

[90] K. Nagao , Y. Nagata , A. Sakuda , et al., “A Reversible Oxygen Redox Reaction in Bulk‐Type All‐Solid‐State Batteries,” Science Advances 6, no. 25 (2020): aax7236. 10.1126/sciadv.aax7236 PMC7304969 32596439

[91] K. Hikima , K. Shimizu , H. Kiuchi , et al., “Reaction Mechanism of Li_2_MnO_3_ Electrodes in an All‐Solid‐State Thin‐Film Battery Analyzed by Operando Hard X‐Ray Photoelectron Spectroscopy,” Journal of the American Chemical Society 144, no. 1 (2022): 236–247. 34957828 10.1021/jacs.1c09087

[92] K. Hikima , Y. Hinuma , K. Shimizu , et al., “Reactions of the Li_2_MnO_3_ Cathode in an All‐Solid‐State Thin‐Film Battery during Cycling,” ACS Applied Materials & Interfaces 13, no. 6 (2021): 7650–7663. 33535741 10.1021/acsami.0c18030

[93] S. Lee , D. Lee , and A. Manthiram , “Mixed Ionic‐Electronic Conductivity of High‐Nickel, Single‐Crystal Cathodes Influencing the Cycling Stability of All‐solid‐State Lithium‐Ion Batteries,” Journal of Materials Chemistry A 12 (2024): 26244–26252, 10.1039/D4TA03727H.

[94] H. Komatsu , S. Banerjee , M. L. Holekevi Chandrappa , et al., “Interfacial Stability of Layered LiNi* _x_ *Mn* _y_ *Co_1–_ * _x_ *—* _y_ *O_2_ Cathodes with Sulfide Solid Electrolytes in All‐Solid‐State Rechargeable Lithium‐Ion Batteries from First‐Principles Calculations,” Journal of Physical Chemistry C 126, no. 41 (2022): 17482–17489, 10.1021/acs.jpcc.2c05336.

[95] S. Wang , M. Yan , Y. Li , C. Vinado , and J. Yang , “Separating Electronic and Ionic Conductivity in Mix‐Conducting Layered Lithium Transition‐Metal Oxides,” Journal of Power Sources 393 (2018): 75–82, 10.1016/j.jpowsour.2018.05.005.

[96] E. Quemin , R. Dugas , A. Chaupatnaik , et al., “An Advanced Cell for Measuring In Situ Electronic Conductivity Evolutions in All‐Solid‐State Battery Composites,” Advanced Energy Materials 13, no. 31 (2023): 2301105.

[97] R. Yu , C. Wang , H. Duan , et al., “Manipulating Charge‐Transfer Kinetics of Lithium‐Rich Layered Oxide Cathodes in Halide All‐Solid‐State Batteries,” Advanced Materials 35 (2022): 2207234, 10.1002/adma.202207234. 36461688

[98] D. H. Kim , D. Y. Oh , K. H. Park , et al., “Infiltration of Solution‐Processable Solid Electrolytes into Conventional Li‐Ion‐Battery Electrodes for All‐Solid‐State Li‐Ion Batteries,” Nano Letters 17, no. 5 (2017): 3013–3020, 10.1021/acs.nanolett.7b00330. 28362097

[99] J. Sung , J. Heo , D.‐H. Kim , et al., “Infiltration‐Driven Performance Enhancement of Poly‐Crystalline Cathodes in All‐Solid‐State Batteries,” NPG Asia Materials 16, no. 1 (2024): 53, 10.1038/s41427-024-00555-7.

[100] P. Minnmann , L. Quillman , S. Burkhardt , F. H. Richter , and J. Janek , “Editors′ Choice—Quantifying the Impact of Charge Transport Bottlenecks in Composite Cathodes of All‐Solid‐State Batteries,” Journal of the Electrochemical Society 168, no. 4 (2021): 040537.

[101] Y. Xiao , Y. Wang , S.‐H. Bo , J. C. Kim , L. J. Miara , and G. Ceder , “Understanding Interface Stability in Solid‐State Batteries,” Nature Reviews Materials 5, no. 2 (2020): 105–126.

[102] K. Takada , T. Ohno , N. Ohta , T. Ohnishi , and Y. Tanaka , “Positive and Negative Aspects of Interfaces in Solid‐State Batteries,” ACS Energy Letters 3, no. 1 (2018): 98–103.

[103] K. Takada , N. Ohta , L. Zhang , et al., “Interfacial Phenomena in Solid‐State Lithium Battery with Sulfide Solid Electrolyte,” Solid State Ionics 225 (2012): 594–597, 10.1016/j.ssi.2012.01.009.

[104] J. Haruyama , K. Sodeyama , L. Han , K. Takada , and Y. Tateyama , “Space–Charge Layer Effect at Interface between Oxide Cathode and Sulfide Electrolyte in All‐Solid‐State Lithium‐Ion Battery,” Chemistry of Materials 26, no. 14 (2014): 4248–4255.

[105] L. Wang , R. Xie , B. Chen , et al., “In Situ Visualization of the Space‐Charge‐Layer Effect on Interfacial Lithium‐Ion Transport in All‐Solid‐State Batteries,” Nature Communications 11, no. 1 (2020): 5889. 10.1038/s41467-020-19726-5 PMC7674427 33208730

[106] L. Zhou , A. Assoud , Q. Zhang , X. Wu , and L. F. Nazar , “New Family of Argyrodite Thioantimonate Lithium Superionic Conductors,” Journal of the American Chemical Society 141, no. 48 (2019): 19002–19013, 10.1021/jacs.9b08357. 31642663

[107] M. A. Kraft , S. Ohno , T. Zinkevich , et al., “Inducing High Ionic Conductivity in the Lithium Superionic Argyrodites Li_6+_ * _x_ *P_1–_ * _x_ *Ge* _x_ *S_5_I for All‐Solid‐State Batteries,” Journal of the American Chemical Society 140, no. 47 (2018): 16330–16339, 10.1021/jacs.8b10282. 30380843

[108] F. Wu , W. Fitzhugh , L. Ye , J. Ning , and X. Li , “Advanced Sulfide Solid Electrolyte by Core‐Shell Structural Design,” Nature Communications 9, no. 1 (2018): 4037. 10.1038/s41467-018-06123-2 PMC6168527 30279498

[109] J. Auvergniot , A. Cassel , J.‐B. Ledeuil , V. Viallet , V. Seznec , and R. Dedryvère , “Interface Stability of Argyrodite Li_6_PS_5_Cl toward LiCoO_2_, LiNi_1/3_Co_1/3_Mn_1/3_O_2_, and LiMn_2_O_4_ in Bulk All‐Solid‐State Batteries,” Chemistry of Materials 29, no. 9 (2017): 3883–3890.

[110] A. Banerjee , H. Tang , X. Wang , et al., “Revealing Nanoscale Solid–Solid Interfacial Phenomena for Long‐Life and High‐Energy All‐Solid‐State Batteries,” ACS Applied Materials & Interfaces 11, no. 46 (2019): 43138–43145, 10.1021/acsami.9b13955. 31642661

[111] R. Koerver , I. Aygün , T. Leichtweiß , et al., “Capacity Fade in Solid‐State Batteries: Interphase Formation and Chemomechanical Processes in Nickel‐Rich Layered Oxide Cathodes and Lithium Thiophosphate Solid Electrolytes,” Chemistry of Materials 29, no. 13 (2017): 5574–5582, 10.1021/acs.chemmater.7b00931.

[112] K. Kim , T. Kim , G. Song , et al., “Trimethylsilyl Compounds for the Interfacial Stabilization of Thiophosphate‐Based Solid Electrolytes in All‐Solid‐State Batteries,” Advanced Science (Weinh) 10, no. 33 (2023): 2303308. 10.1002/advs.202303308 PMC10667807 37867236

[113] S. Lee , T. Kim , K. Kim , et al., “Mechano‐Electrochemical Healing at the Interphase between LiNi_0.8_Co_0.1_Mn_0.1_O_2_ and Li_6_PS_5_Cl in All‐Solid‐State Batteries,” Advanced Energy Materials 15 (2025): 2405782, 10.1002/aenm.202405782.

[114] A. Hayashi , S. Hama , T. Minami , and M. Tatsumisago , “Formation of Superionic Crystals from Mechanically Milled Li_2_S–P_2_S_5_ Glasses,” Electrochemistry Communications 5, no. 2 (2003): 111–114, 10.1016/S1388-2481(02)00555-6.

[115] A. Sakuda , A. Hayashi , and M. Tatsumisago , “Sulfide Solid Electrolyte with Favorable Mechanical Property for All‐Solid‐State Lithium Battery,” Scientific Reports 3, no. 1 (2013): 2261, 10.1038/srep02261. 23877241 PMC3719077

[116] N. Kamaya , K. Homma , Y. Yamakawa , et al., “A Lithium Superionic Conductor,” Nature Materials 10, no. 9 (2011): 682–686, 10.1038/nmat3066. 21804556

[117] H.‐J. Deiseroth , S.‐T. Kong , H. Eckert , et al., “Li_6_PS_5_X: A Class of Crystalline Li‐Rich Solids with an Unusually High Li^+^ Mobility,” Angewandte Chemie International Edition 47, no. 4 (2008): 755–758, 10.1002/anie.200703900. 18161703

[118] S.‐T. Kong , H.‐J. Deiseroth , C. Reiner , et al., “Lithium Argyrodites with Phosphorus and Arsenic: Order and Disorder of Lithium Atoms, Crystal Chemistry, and Phase Transitions,” Chemistry European Journal 16, no. 7 (2010): 2198–2206. 10.1002/chem.200902470 20066696

[119] K. Jun , Y. Chen , G. Wei , X. Yang , and G. Ceder , “Diffusion Mechanisms of Fast Lithium‐Ion Conductors,” Nature Reviews Materials 9, no. 12 (2024): 887–905.

[120] Y. Wang , W. D. Richards , S. P. Ong , et al., “Design Principles for Solid‐State Lithium Superionic Conductors,” Nature Materials 14, no. 10 (2015): 1026–1031, 10.1038/nmat4369. 26280225

[121] R. Schlem , M. Ghidiu , S. P. Culver , A.‐L. Hansen , and W. G. Zeier , “Changing the Static and Dynamic Lattice Effects for the Improvement of the Ionic Transport Properties within the Argyrodite Li_6_PS_5–_ * _x_ *Se* _x_ *I,” ACS Appl Energy Mater 3, no. 1 (2020): 9–18.

[122] S. V. Patel , S. Banerjee , H. Liu , et al., “Tunable Lithium‐Ion Transport in Mixed‐Halide Argyrodites Li_6–_ * _x_ *PS_5–_ * _x_ *ClBr* _x_ *: An Unusual Compositional Space,” Chemistry of Materials 33, no. 4 (2021): 1435–1443.

[123] H.‐J. Deiseroth , J. Maier , K. Weichert , V. Nickel , S.‐T. Kong , and C. Reiner , “Li_7_PS_6_ and Li_6_PS_5_ *X*(X: Cl, Br, I): Possible Three‐Dimensional Diffusion Pathways for Lithium Ions and Temperature Dependence of the Ionic Conductivity by Impedance Measurements,” Zeitschrift für Anorganische und Allgemeine Chemie 637, no. 10 (2011): 1287–1294, 10.1002/zaac.201100158.

[124] E. Zhao , L. He , Z. Zhang , et al., “New Insights into Li Distribution in the Superionic Argyrodite Li_6_PS_5_Cl,” Chemical Communications 57, no. 82 (2021): 10787–10790. 34590100 10.1039/d1cc03083c

[125] C. Yu , S. Ganapathy , E. R. H. van Eck , et al., “Revealing the Relation between the Structure, Li‐Ion Conductivity and Solid‐State Battery Performance of the Argyrodite Li_6_PS_5_Br Solid Electrolyte,” Journal of Materials Chemistry A 5, no. 40 (2017): 21178–21188, 10.1039/C7TA05031C.

[126] M. A. Kraft , S. P. Culver , M. Calderon , et al., “Influence of Lattice Polarizability on the Ionic Conductivity in the Lithium Superionic Argyrodites Li_6_PS_5_X (X = Cl, Br, I),” Journal of the American Chemical Society 139, no. 31 (2017): 10909–10918, 10.1021/jacs.7b06327. 28741936

[127] N. J. J. de Klerk , I. Rosłoń , and M. Wagemaker , “Diffusion Mechanism of Li Argyrodite Solid Electrolytes for Li‐Ion Batteries and Prediction of Optimized Halogen Doping: the Effect of Li Vacancies, Halogens, and Halogen Disorder,” Chemistry of Materials 28, no. 21 (2016): 7955–7963, 10.1021/acs.chemmater.6b03630.

[128] P. Adeli , J. D. Bazak , A. Huq , G. R. Goward , and L. F. Nazar , “Influence of Aliovalent Cation Substitution and Mechanical Compression on Li‐Ion Conductivity and Diffusivity in Argyrodite Solid Electrolytes,” Chemistry of Materials 33, no. 1 (2021): 146–157.

[129] J. Zhang , L. Li , C. Zheng , et al., “Silicon‐Doped Argyrodite Solid Electrolyte Li_6_PS_5_I with Improved Ionic Conductivity and Interfacial Compatibility for High‐Performance All‐Solid‐State Lithium Batteries,” ACS Applied Materials & Interfaces 12, no. 37 (2020): 41538–41545, 10.1021/acsami.0c11683. 32822167

[130] D. Lee , Y. Shim , E. Choi , et al., “Alleviating Kinetical Delamination Induced by Localized Cathode Contact via Electrochemo‐Mechanical Modeling in All‐Solid‐State Batteries,” Joule 9, no. 8 (2025): 102046, 10.1016/j.joule.2025.102046.

[131] M.‐G. Jeong , K. G. Naik , Y. Zheng , et al., “Operando Investigation on the Role of Densification and Chemo‐Mechanics on Solid‐State Cathodes,” Advanced Energy Materials 14, no. 23 (2024): 2304544.

[132] E. Schlautmann , A. Weiß , O. Maus , et al., “Impact of the Solid Electrolyte Particle Size Distribution in Sulfide‐Based Solid‐State Battery Composites,” Advanced Energy Materials 13, no. 41 (2023): 2302309.

[133] Z. Liu , W. Fu , E. A. Payzant , et al., “Anomalous High Ionic Conductivity of Nanoporous β‐Li_3_PS_4_ ,” Journal of the American Chemical Society 135, no. 3 (2013): 975–978, 10.1021/ja3110895. 23305294

[134] A. Miura , N. C. Rosero‐Navarro , A. Sakuda , et al., “Liquid‐Phase Syntheses of Sulfide Electrolytes for All‐Solid‐State Lithium Battery,” Nature Reviews Chemistry 3, no. 3 (2019): 189–198, 10.1038/s41570-019-0078-2.

[135] J. Xu , L. Liu , N. Yao , F. Wu , H. Li , and L. Chen , “Liquid‐Involved Synthesis and Processing of Sulfide‐Based Solid Electrolytes, Electrodes, and All‐Solid‐State Batteries,” Materials Today Nano 8 (2019): 100048, 10.1016/j.mtnano.2019.100048.

[136] L. Zhou , K.‐H. Park , X. Sun , et al., “Solvent‐Engineered Design of Argyrodite Li_6_PS_5_X (X = Cl, Br, I) Solid Electrolytes with High Ionic Conductivity,” ACS Energy Letters 4, no. 1 (2019): 265–270, 10.1021/acsenergylett.8b01997.

[137] D. Kim , S.‐H. Hwang , S.‐D. Seo , H. Yeo , and D.‐W. Kim , “Size‐Regulated Li‐Argyrodite Particles via Wet‐Chemical Route for Enhanced Solid‐State Battery Performance,” Chemical Engineering Journal 509 (2025): 161415, 10.1016/j.cej.2025.161415.

[138] Y. Huh , H. Gon Lee , C.‐M. Cho , et al., “Solution‐Processed Synthesis of Nano‐Sized Argyrodite Solid Electrolytes with Cavitation Effect for High Performance All‐Solid‐State Lithium‐Ion Batteries,” Batteries & Supercaps 6, no. 4 (2023): 202300036.

[139] N. C. Rosero‐Navarro , A. Miura , and K. Tadanaga , “Composite Cathode Prepared by Argyrodite Precursor Solution Assisted by Dispersant Agents for Bulk‐Type All‐Solid‐State Batteries,” Journal of Power Sources 396 (2018): 33–40, 10.1016/j.jpowsour.2018.06.011.

[140] S.‐M. Bak , E. Hu , Y. Zhou , et al., “Structural Changes and Thermal Stability of Charged LiNi* _x_ *Mn* _y_ *Co* _Z_ *O_2_ Cathode Materials Studied by Combined *In Situ* Time‐Resolved XRD and Mass Spectroscopy Combined In Situ Time‐Resolved XRD and Mass Spectroscopy,” ACS Applied Materials & Interfaces 6, no. 24 (2014): 22594–22601, 10.1021/am506712c. 25420188

[141] T. Bartsch , F. Strauss , T. Hatsukade , et al., “Gas Evolution in All‐Solid‐State Battery Cells,” ACS Energy Letters 3, no. 10 (2018): 2539–2543.

[142] F. Strauss , J. H. Teo , A. Schiele , et al., “Gas Evolution in Lithium‐Ion Batteries: Solid versus Liquid Electrolyte,” ACS Applied Materials & Interfaces 12, no. 18 (2020): 20462–20468. 32275815 10.1021/acsami.0c02872

[143] Y.‐T. Chen , M. A. T. Marple , D. H. S. Tan , et al., “Investigating Dry Room Compatibility of Sulfide Solid‐State Electrolytes for Scalable Manufacturing,” Journal of Materials Chemistry A 10, no. 13 (2022): 7155–7164, 10.1039/D1TA09846B.

[144] K. Yoon , H. Kim , S. Han , et al., “Detrimental Effect of High‐Temperature Storage on Sulfide‐Based All‐Solid‐State Batteries,” Applied Physics Reviews 9, no. 3 (2022): 031403.

[145] H. Lee , J. Seok , C. Chung , S. Park , J. Kim , and W.‐S. Yoon , “Impact of High‐Temperature Storage on Capacity Fading of Ni‐Rich Cathodes in Sulfide‐Based All‐Solid‐State Batteries,” Chemical Engineering Journal 498 (2024): 154903, 10.1016/j.cej.2024.154903.

[146] J. Park , K. Kim , S. Lee , et al., “Mechano‐Electrochemical and Structural Insights into Composition‐Driven Behavior of LiNi_1‐x‐y_Co_x_Mn_y_O_2_(0.3 < 1‐x‐y ≤ 0.8) Cathodes in Sulfide‐Based All‐Solid‐State Batteries,” Advanced Functional Materials (2025): 17263.

[147] C. Zhan , T. Wu , J. Lu , and K. Amine , “Dissolution, Migration, and Deposition of Transition Metal Ions in Li‐Ion Batteries Exemplified by Mn‐Based Cathodes—A Critical Review,” Energy & Environmental Science 11, no. 2 (2018): 243–257.

[148] R. Zhang , F. Strauss , L. Jiang , et al., “Transition‐Metal Interdiffusion and Solid Electrolyte Poisoning in All‐Solid‐State Batteries Revealed by Cryo‐TEM,” Chemical Communications 59, no. 31 (2023): 4600–4603. 36920228 10.1039/d3cc00516j

[149] A. Sakuda , A. Hayashi , and M. Tatsumisago , “Interfacial Observation between LiCoO_2_ Electrode and Li_2_S−P_2_S_5_ Solid Electrolytes of all‐Solid‐State Lithium Secondary Batteries Using Transmission Electron Microscopy,” Chemistry of Materials 22, no. 3 (2010): 949–956, 10.1021/cm901819c.

[150] A. M. Stavola , E. K. Zimmerer , X. Sun , et al., “Detection of a Cobalt‐Containing Interphase at the Li_6_PS_5_Cl‐NMC111 Interface by in Situ *μ*XANES and EIS,” Journal of the Electrochemical Society 171, no. 3 (2024): 030501.

[151] F. Walther , F. Strauss , X. Wu , et al., “The Working Principle of a Li_2_CO_3_/LiNbO_3_ Coating on NCM for Thiophosphate‐Based All‐Solid‐State Batteries,” Chemistry of Materials 33, no. 6 (2021): 2110–2125.

[152] F. Strauss , J. H. Teo , J. Maibach , et al., “Li_2_ZrO_3_‐Coated NCM622 for Application in Inorganic Solid‐State Batteries: Role of Surface Carbonates in the Cycling Performance,” ACS Applied Materials & Interfaces 12, no. 51 (2020): 57146–57154. 33302618 10.1021/acsami.0c18590

[153] Y. Morino , H. Tsukasaki , and S. Mori , “Microscopic Degradation Mechanism of Argyrodite‐Type Sulfide at the Solid Electrolyte–Cathode Interface,” ACS Applied Materials & Interfaces 15, no. 19 (2023): 23051–23057. 37130265 10.1021/acsami.3c00462

[154] Y.‐Q. Zhang , Y. Tian , Y. Xiao , et al., “Direct Visualization of the Interfacial Degradation of Cathode Coatings in Solid State Batteries: A Combined Experimental and Computational Study,” Advanced Energy Materials 10, no. 27 (2020): 1903778.

[155] S. Kobayashi , H. Watanabe , T. Kato , F. Mizuno , and A. Kuwabara , “Atomic‐Scale Observations of Oxygen Release Degradation in Sulfide‐Based All‐Solid‐State Batteries with Layered Oxide Cathodes,” ACS Applied Materials & Interfaces 14, no. 34 (2022): 39459. 35981095 10.1021/acsami.2c06950

[156] C. Park , J. Lee , S. Lee , Y. J. Han , J. Kim , and S.‐K. Jung , “Organic‐Additive‐Derived Cathode Electrolyte Interphase Layer Mitigating Intertwined Chemical and Mechanical Degradation for Sulfide‐Based Solid‐State Batteries,” Advanced Energy Materials 13 (2023): 2203861, 10.1002/aenm.202203861.

[157] B.‐X. Shi , Y. Yusim , S. Sen , et al., “Mitigating Contact Loss in Li_6_PS_5_ Cl‐Based Solid‐State Batteries Using a Thin Cationic Polymer Coating on NCM,” Advanced Energy Materials 13 (2023): 2300310, 10.1002/aenm.202300310.

[158] Y. Li , S. Daikuhara , S. Hori , et al., “Oxygen Substitution for Li–Si–P–S–Cl Solid Electrolytes toward Purified Li_10_GeP_2_S_12_‐Type Phase with Enhanced Electrochemical Stabilities for All‐Solid‐State Batteries,” Chemistry of Materials 32, no. 20 (2020): 8860–8867, 10.1021/acs.chemmater.0c02351.

[159] T. Ohtomo , F. Mizuno , A. Hayashi , K. Tadanaga , and M. Tatsumisago , “Electrical and Electrochemical Properties of Li_2_S–P_2_S_5_–P_2_O_5_ Glass–Ceramic Electrolytes,” Journal of Power Sources 146, no. 1 (2005): 715–718, 10.1016/j.jpowsour.2005.03.063.

[160] K.‐H. Kim and S. W. Martin , “Structures and Properties of Oxygen‐Substituted Li_10_SiP_2_S_12–_ * _x_ *O* _x_ * Solid‐State Electrolytes,” Chemistry of Materials 31, no. 11 (2019): 3984–3991, 10.1021/acs.chemmater.9b00505.

[161] T. T. Zuo , F. Walther , J. H. Teo , et al., “Impact of the Chlorination of Lithium Argyrodites on the Electrolyte/Cathode Interface in Solid‐State Batteries,” Angewandte Chemie (International Ed in English) 62, no. 7 (2023): 202213228. 10.1002/anie.202213228 PMC10107527 36416271

[162] W. Zhang , T. Leichtweiss , S. P. Culver , et al., “The Detrimental Effects of Carbon Additives in Li_10_GeP_2_S_12_‐Based Solid‐State Batteries,” ACS Applied Materials & Interfaces 9, no. 41 (2017): 35888–35896. 28937736 10.1021/acsami.7b11530

[163] F. Walther , S. Randau , Y. Schneider , et al., “Influence of Carbon Additives on the Decomposition Pathways in Cathodes of Lithium Thiophosphate‐Based All‐Solid‐State Batteries,” Chemistry of Materials 32, no. 14 (2020): 6123–6136, 10.1021/acs.chemmater.0c01825.

[164] D. H. S. Tan , E. A. Wu , H. Nguyen , et al., “Elucidating Reversible Electrochemical Redox of Li_6_PS_5_Cl Solid Electrolyte,” ACS Energy Letters 4, no. 10 (2019): 2418–2427, 10.1021/acsenergylett.9b01693.

[165] T. K. Schwietert , V. A. Arszelewska , C. Wang , et al., “Clarifying the Relationship between Redox Activity and Electrochemical Stability in Solid Electrolytes,” Nature Materials 19, no. 4 (2020): 428–435, 10.1038/s41563-019-0576-0. 31932670

[166] M. Liu , J. J. Hong , E. Sebti , et al., “Surface Molecular Engineering to Enable Processing of Sulfide Solid Electrolytes in Humid Ambient Air,” Nature Communications 16, no. 1 (2025): 213. 10.1038/s41467-024-55634-8 PMC11696013 39747166

[167] Z. D. Hood , A. U. Mane , A. Sundar , et al., “Multifunctional Coatings on Sulfide‐Based Solid Electrolyte Powders with Enhanced Processability, Stability, and Performance for Solid‐State Batteries,” Advanced Materials 35, no. 21 (2023): 2300673. 10.1002/adma.202300673 36929566

[168] K. T. Kim , J.‐S. Kim , K. H. Baeck , et al., “Surface Fluorination Shielding of Sulfide Solid Electrolytes for Enhanced Electrochemical Stability in All‐Solid‐State Batteries,” Advanced Materials 37, no. 35 (2025): 2416816, 10.1002/adma.202416816. 40545876

[169] S. Wang , M. Tang , Q. Zhang , et al., “Lithium Argyrodite as Solid Electrolyte and Cathode Precursor for Solid‐State Batteries with Long Cycle Life,” Advanced Energy Materials 11, no. 31 (2021): 2101370.

[170] J. Jang , Y.‐T. Chen , G. Deysher , et al., “Enabling a Co‐Free, High‐Voltage LiNi_0.5_Mn_1.5_O_4_ Cathode in All‐Solid‐State Batteries with a Halide Electrolyte,” ACS Energy Letters 7, no. 8 (2022): 2531–2539.

[171] K. Kim , S. Jun , T. Kim , et al., “Interfacial Degradation Mechanism of Nanostructured LiCoO_2_ for Li_6_PS_5_Cl‐Based All‐Solid‐State Batteries,” Chemistry of Materials 36, no. 10 (2024): 5215–5227.

[172] H. Kim , G. Choi , S. Kim , et al., “Plane‐Selective Coating of Li_2_SnO_3_ on Li[NixCo_1–x_]O_2_ for High Power Li Ion Batteries,” Journal of Physical Chemistry Letters 11, no. 17 (2020): 7096–7102. 32787329 10.1021/acs.jpclett.0c01829

[173] G. Liu , J. Shi , M. Zhu , et al., “Ultra‐Thin Free‐Standing Sulfide Solid Electrolyte Film for Cell‐Level High Energy Density All‐Solid‐State Lithium Batteries,” Energy Storage Materials 38 (2021): 249–254, 10.1016/j.ensm.2021.03.017.

[174] S. Puls , E. Nazmutdinova , F. Kalyk , et al., “Benchmarking the Reproducibility of All‐Solid‐State Battery Cell Performance,” Nature Energy 9 (2024): 1310–1320, 10.1038/s41560-024-01634-3.

[175] J. Schnell , T. Günther , T. Knoche , et al., “All‐Solid‐State Lithium‐Ion and Lithium Metal Batteries—Paving the Way to Large‐Scale Production,” Journal of Power Sources 382 (2018): 160–175.

[176] L. Fernandez‐Diaz , J. Castillo , E. Sasieta‐Barrutia , et al., “Mixing Methods for Solid State Electrodes: Techniques, Fundamentals, Recent Advances, and Perspectives,” Chemical Engineering Journal 464 (2023): 142469, 10.1016/j.cej.2023.142469.

[177] Y. J. Kim , T. D. Hoang , S. C. Han , et al., “Exploring Optimal Cathode Composite Design for High‐Performance All‐Solid‐State Batteries,” Energy Storage Materials 71 (2024): 103607, 10.1016/j.ensm.2024.103607.

[178] M. Kissel , M. Schosland , J. Töws , et al., “Quantifying the Impact of Cathode Composite Mixing Quality on Active Mass Utilization and Reproducibility of Solid‐State Battery Cells,” Advanced Energy Materials 15, no. 27 (2025): 2405405.

[179] Z. Liang , Y. Xiao , K. Wang , et al., “Enabling Stable and High Areal Capacity Solid State Battery with Ni‐Rich Cathode via Failure Mechanism Study,” Energy Storage Materials 63 (2023): 102987, 10.1016/j.ensm.2023.102987.

[180] K. Raju , L. Wheatcroft , M. C. Lai , et al., “Influence of Cathode Calendering Density on the Cycling Stability of Li‐Ion Batteries Using NMC811 Single or Poly Crystalline Particles,” Journal of the Electrochemical Society 171, no. 8 (2024): 080519.

[181] H. Kim , S. K. Oh , J. Lee , S. W. Doo , Y. Kim , and K. T. Lee , “Failure Mode of Thick Cathodes for Li‐Ion Batteries: Variation of State‐of‐Charge Along the Electrode Thickness Direction,” Electrochimica Acta 370 (2021): 137743, 10.1016/j.electacta.2021.137743.

[182] F. Jiang and P. Peng , “Elucidating the Performance Limitations of Lithium‐Ion Batteries due to Species and Charge Transport through Five Characteristic Parameters,” Scientific Reports 6, no. 1 (2016): 32639. 27599870 10.1038/srep32639 PMC5013525

[183] J. Schnell , H. Knörzer , A. J. Imbsweiler , and G. Reinhart , “Solid versus Liquid—A Bottom‐Up Calculation Model to Analyze the Manufacturing Cost of Future High‐Energy Batteries,” Energy Technology 8, no. 3 (2020): 1901237, 10.1002/ente.201901237.

[184] D. H. S. Tan , Y. S. Meng , and J. Jang , “Scaling Up High‐Energy‐Density Sulfidic Solid‐State Batteries: A Lab‐to‐Pilot Perspective,” Joule 6, no. 8 (2022): 1755–1769.

[185] T. Ji , Y. Zhang , J. Torres , et al., “Operando Neutron Imaging‐Guided Gradient Design of Li‐Ion Solid Conductor for High‐Mass‐Loading Cathodes,” Nature Communications 16, no. 1 (2025): 7667. 10.1038/s41467-025-62518-y PMC12361399 40825937

[186] A. M. Stavola , X. Sun , D. P. Guida , et al., “Lithiation Gradients and Tortuosity Factors in Thick NMC111‐Argyrodite Solid‐State Cathodes,” ACS Energy Letters 8, no. 2 (2023): 1273–1280. 37941794 10.1021/acsenergylett.2c02699 PMC10629242

[187] H. Shen , S. Jing , S. Liu , et al., “Tailoring the Electronic Conductivity of High‐Loading Cathode Electrodes for Practical Sulfide‐Based All‐Solid‐State Batteries,” Advanced Powder Materials 2, no. 4 (2023): 100136.

[188] E. Schlautmann , J. Drews , L. Ketter , et al., “Graded Cathode Design for Enhanced Performance of Sulfide‐Based Solid‐State Batteries,” ACS Energy Letters 10, no. 4 (2025): 1664–1670.

[189] J. Liang , K. Qian , C. Xiao , et al., “Longitudinal Spatial Charge Transfer Optimization in Composite Cathodes Enables Ultra‐Stable All‐Solid‐State Batteries,” Energy & Environmental Science 18, no. 18 (2025): 8564–8574.

[190] Y.‐T. Chen , J. Jang , J. A. S. Oh , et al., “Enabling Uniform and Accurate Control of Cycling Pressure for All‐Solid‐State Batteries,” Advanced Energy Materials 14, no. 30 (2024): 2304327, 10.1002/aenm.202304327.

[191] K. Lee , S. Kim , J. Park , et al., “Selection of Binder and Solvent for Solution‐Processed All‐Solid‐State Battery,” Journal of The Electrochemical Society 164, no. 9 (2017): A2075–A2081, 10.1149/2.1341709jes.

[192] A. Bielefeld , D. A. Weber , and J. Janek , “Modeling Effective Ionic Conductivity and Binder Influence in Composite Cathodes for all‐Solid‐State Batteries,” ACS Applied Materials & Interfaces 12, no. 11 (2020): 12821–12833, 10.1021/acsami.9b22788. 32093477

[193] J. H. Teo , F. Strauss , F. Walther , et al., “The Interplay between (electro)Chemical and (chemo)Mechanical Effects in the Cycling Performance of Thiophosphate‐Based Solid‐State Batteries,” Materials Futures 1, no. 1 (2022): 015102.

[194] J. Kim , W. Lee , J. Seok , et al., “Critical Factors to Understanding the Electrochemical Performance of All‐Solid‐State Batteries: Solid Interfaces and Non‐Zero Lattice Strain,” Small 19, no. 42 (2023): 2304269. 10.1002/smll.202304269 37317038

[195] M. Yamamoto , Y. Terauchi , A. Sakuda , and M. Takahashi , “Binder‐Free Sheet‐Type All‐Solid‐State Batteries with Enhanced Rate Capabilities and High Energy Densities,” Scientific Reports 8, no. 1 (2018): 1212, 10.1038/s41598-018-19398-8. 29352273 PMC5775432

[196] P. Karanth , M. Weijers , P. Ombrini , D. Ripepi , F. Ooms , and F. M. Mulder , “A Phase Inversion Strategy for Low‐Tortuosity and Ultrahigh‐Mass‐Loading Nickel‐Rich Layered Oxide Electrodes,” Cell Reports Physical Science 5, no. 6 (2024): 101972, 10.1016/j.xcrp.2024.101972.

[197] X. Wang , L. Ye , C.‐W. Nan , and X. Li , “Effect of Solvents on a Li_10_GeP_2_S_12_‐Based Composite Electrolyte via Solution Method for Solid‐State Battery Applications,” ACS Applied Materials & Interfaces 14, no. 41 (2022): 46627–46634. 36197083 10.1021/acsami.2c12920

[198] J. Ruhl , L. M. Riegger , M. Ghidiu , and W. G. Zeier , “Impact of Solvent Treatment of the Superionic Argyrodite Li_6_PS_5_Cl on Solid‐State Battery Performance,” Advanced Energy and Sustainablility Research 2, no. 2 (2021): 2000077.

[199] K. Lee , J. Lee , S. Choi , K. Char , and J. W. Choi , “Thiol–Ene Click Reaction for Fine Polarity Tuning of Polymeric Binders in Solution‐Processed All‐Solid‐State Batteries,” ACS Energy Letters 4, no. 1 (2019): 94–101, 10.1021/acsenergylett.8b01726.

[200] T. Y. Kwon , K. T. Kim , D. Y. Oh , Y. B. Song , S. Jun , and Y. S. Jung , “Three‐Dimensional Networking Binders Prepared In Situ during Wet‐Slurry Process for All‐Solid‐State Batteries Operating under Low External Pressure,” Energy Storage Materials 49 (2022): 219–226.

[201] Y. Li , X. Wang , H. Zhou , et al., “Thin Solid Electrolyte Layers Enabled by Nanoscopic Polymer Binding,” ACS Energy Letters 5, no. 3 (2020): 955–961.

[202] W.‐H. Jeong , H. Kim , S. Kansara , et al., “Stimulating the Electrostatic Interactions in Composite Cathodes Using a Slurry‐Fabricable Polar Binder for Practical All‐Solid‐State Batteries,” Energy Storage Materials 73 (2024): 103855.

[203] M.‐J. Kim , J.‐W. Park , B. G. Kim , et al., “Facile Fabrication of Solution‐Processed Solid‐Electrolytes for High‐Energy‐Density All‐Solid‐State‐Batteries by Enhanced Interfacial Contact,” Scientific Reports 10, no. 1 (2020): 11923, 10.1038/s41598-020-68885-4. 32681025 PMC7367834

[204] K. T. Kim , D. Y. Oh , S. Jun , et al., “Tailoring Slurries Using Cosolvents and Li Salt Targeting Practical All‐Solid‐State Batteries Employing Sulfide Solid Electrolytes,” Advanced Energy Materials 11, no. 17 (2021): 2003766.

[205] S. Jing , H. Shen , Y. Huang , et al., “Toward the Practical and Scalable Fabrication of Sulfide‐Based All‐Solid‐State Batteries: Exploration of Slurry Process and Performance Enhancement via the Addition of LiClO_4_ ,” Advanced Functional Materials 33, no. 24 (2023): 2214274.

[206] F. Hippauf , B. Schumm , S. Doerfler , et al., “Overcoming Binder Limitations of Sheet‐Type Solid‐State Cathodes Using a Solvent‐Free Dry‐Film Approach,” Energy Storage Materials 21 (2019): 390–398, 10.1016/j.ensm.2019.05.033.

[207] B. Schumm , A. Dupuy , M. Lux , et al., “Dry Battery Electrode Technology: From Early Concepts to Industrial Applications,” Advanced Energy Materials 15, no. 24 (2025): 2406011.

[208] Y. Lu , C.‐Z. Zhao , H. Yuan , J.‐K. Hu , J.‐Q. Huang , and Q. Zhang , “Dry Electrode Technology, the Rising Star in Solid‐State Battery Industrialization,” Matter 5, no. 3 (2022): 876–898.

[209] H. Sul , D. Lee , and A. Manthiram , “High‐Loading Lithium‐Sulfur Batteries with Solvent‐Free Dry‐Electrode Processing,” Small 20, no. 31 (2024): 2400728. 10.1002/smll.202400728 38433393

[210] J. Hong , J. Yoon , J.‐W. Park , Y.‐C. Ha , J. Lee , and I. Hwang , “Optimization of PTFE Fibrillation in Dry Electrode Process for Scalable All‐Solid‐State Battery Manufacturing,” Journal of Power Sources 655 (2025): 237925, 10.1016/j.jpowsour.2025.237925.

[211] K.‐H. Lee , H. Shim , S. H. Lee , et al., “Dual‐Fibrous PTFE Structure Enabling Uniform and Thick Dry Electrodes for High‐Energy‐Density and Long‐Lasting Batteries,” Energy & Environmental Science 18, no. 18 (2025): 8446–8461.

[212] D. Lee , Y. Shim , Y. Kim , et al., “Shear Force Effect of the Dry Process on Cathode Contact Coverage in All‐Solid‐State Batteries,” Nature Communications 15, no. 1 (2024): 4763. 10.1038/s41467-024-49183-3 PMC11150245 38834619

[213] D. J. Lee , J. Jang , J.‐P. Lee , et al., “Physio‐Electrochemically Durable Dry‐Processed Solid‐State Electrolyte Films for All‐Solid‐State Batteries,” Advanced Functional Materials 33, no. 28 (2023): 2301341, 10.1002/adfm.202301341.

[214] K. Lee , Y. Jo , J. Seok Nam , H. Yu , and Y.‐J. Kim , “Dry‐Film Technology Employing Cryo‐Pulverized Polytetrafluoroethylene Binder for All‐Solid‐State Batteries,” Chemical Engineering Journal 487 (2024): 150221.

[215] S. Ohno , T. Bernges , J. Buchheim , et al., “How Certain Are the Reported Ionic Conductivities of Thiophosphate‐Based Solid Electrolytes? An Interlaboratory Study,” ACS Energy Letters 5, no. 3 (2020): 910–915.

[216] Y. Sakka , H. Yamashige , A. Watanabe , et al., “Pressure Dependence on the Three‐dimensional Structure of a Composite Electrode in an All‐Solid‐State Battery,” Journal of Materials Chemistry A 10, no. 31 (2022): 16602–16609, 10.1039/D2TA02378D.

[217] J.‐M. Doux , Y. Yang , D. H. S. Tan , et al., “Pressure Effects on Sulfide Electrolytes for All Solid‐State Batteries,” Journal of Materials Chemistry A 8, no. 10 (2020): 5049–5055, 10.1039/C9TA12889A.

[218] C.‐J. Huang , J. A. S. Oh , M. Vicencio , et al., “X‐Ray Micro‐Computed Tomography for Structural Analysis of All‐Solid‐State Battery at Pouch Cell Level,” ACS Energy Letters 10, no. 7 (2025): 3459–3470. 40672128 10.1021/acsenergylett.5c00956 PMC12261322

[219] M. Dixit , C. Beamer , R. Amin , et al., “The Role of Isostatic Pressing in Large‐Scale Production of Solid‐State Batteries,” ACS Energy Letters 7, no. 11 (2022): 3936–3946.

[220] M. Alabdali , F. M. Zanotto , M. Chouchane , et al., “Understanding Mechanical Stresses Upon Solid‐State Battery Electrode Cycling Using Discrete Element Method,” Energy Storage Materials 70 (2024): 103527, 10.1016/j.ensm.2024.103527.

[221] X. Gao , B. Liu , B. Hu , et al., “Solid‐State Lithium Battery Cathodes Operating at Low Pressures,” Joule 6, no. 3 (2022): 636–646.

[222] C. Lee , J. Y. Kim , K. Y. Bae , et al., “Enhancing Electrochemomechanics: How Stack Pressure Regulation Affects All‐Solid‐State Batteries,” Energy Storage Materials 66 (2024): 103196, 10.1016/j.ensm.2024.103196.

[223] T. Shi , Y.‐Q. Zhang , Q. Tu , Y. Wang , M. C. Scott , and G. Ceder , “Characterization of Mechanical Degradation in an All‐Solid‐State Battery Cathode,” Journal of Materials Chemistry A 8, no. 34 (2020): 17399–17404, 10.1039/D0TA06985J.

[224] H.‐J. Shin , J. T. Kim , A. Y. Kim , et al., “New Consideration of Degradation Accelerating of All‐Solid‐State Batteries under a Low‐Pressure Condition,” Advanced Energy Materials 13 (2023): 2301220, 10.1002/aenm.202301220.

[225] K. G. Naik , M. K. Jangid , B. S. Vishnugopi , N. P. Dasgupta , and P. P. Mukherjee , “Interrogating the Role of Stack Pressure in Transport‐Reaction Interaction in the Solid‐State Battery Cathode,” Advanced Energy Materials 15, no. 10: 2403360, 10.1002/aenm.202403360.

[226] A. K. Sharma , B. S. Vishnugopi , A. Ayyaswamy , et al., “Co‐Design of Active Material and Solid Electrolyte Particulate Phases in Solid‐State Battery Composite Electrodes,” ACS Applied Materials & Interfaces 17, no. 35 (2025): 49520–49532. 40842137 10.1021/acsami.5c10908

[227] S. Y. Han , C. Lee , J. A. Lewis , et al., “Stress Evolution During Cycling of Alloy‐Anode Solid‐State Batteries,” Joule 5, no. 9 (2021): 2450–2465.

[228] S. Jun , Y. J. Nam , H. Kwak , K. T. Kim , D. Y. Oh , and Y. S. Jung , “Operando Differential Electrochemical Pressiometry for Probing Electrochemo‐Mechanics in All‐Solid‐State Batteries,” Advanced Functional Materials 30, no. 31 (2020): 2002535, 10.1002/adfm.202002535.

[229] W. Zhang , D. Schröder , T. Arlt , et al., “(Electro)chemical Expansion during Cycling: Monitoring the Pressure Changes in Operating Solid‐State Lithium Batteries,” Journal of Materials Chemistry A 5, no. 20 (2017): 9929–9936, 10.1039/C7TA02730C.

[230] C. Hänsel and D. Kundu , “The Stack Pressure Dilemma in Sulfide Electrolyte Based Li Metal Solid‐State Batteries: A Case Study with Li_6_PS_5_Cl Solid Electrolyte,” Advanced Materials Interfaces 8 (2021): 2100206, 10.1002/admi.202100206.

[231] J.‐M. Doux , H. Nguyen , D. H. S. Tan , et al., “Stack Pressure Considerations for Room‐Temperature All‐Solid‐State Lithium Metal Batteries,” Advanced Energy Materials 10, no. 1 (2020): 1903253, 10.1002/aenm.201903253.

[232] D. Y. Oh , Y. J. Nam , K. H. Park , et al., “Excellent Compatibility of Solvate Ionic Liquids with Sulfide Solid Electrolytes: Toward Favorable Ionic Contacts in Bulk‐Type All‐Solid‐State Lithium‐Ion Batteries,” Advanced Energy Materials 5, no. 22 (2015): 1500865, 10.1002/aenm.201500865.

[233] D. J. Lee , Y. Jeon , J. P. Lee , et al., “Robust Interface and Reduced Operation Pressure Enabled by Co‐Rolling Dry‐Process for Stable All‐Solid‐State Batteries,” Nature Communications 16, no. 1 (2025): 4200. 10.1038/s41467-025-59363-4 PMC12055973 40328773

[234] H. Muramatsu , A. Hayashi , T. Ohtomo , S. Hama , and M. Tatsumisago , “Structural Change of Li_2_S–P_2_S_5_ Sulfide Solid Electrolytes in the Atmosphere,” Solid State Ionics 182, no. 1 (2011): 116–119, 10.1016/j.ssi.2010.10.013.

[235] X. Zhang , X. Li , S. Weng , et al., “Spontaneous Gas–Solid Reaction on Sulfide Electrolytes for High‐Performance All‐Solid‐State Batteries,” Energy & Environmental Science 16, no. 3 (2023): 1091–1099.

[236] Y. Nikodimos , C.‐J. Huang , B. W. Taklu , W.‐N. Su , and B. J. Hwang , “Chemical Stability of Sulfide Solid‐State Electrolytes: Stability toward Humid Air and Compatibility with Solvents and Binders,” Energy & Environmental Science 15, no. 3 (2022): 991–1033.

[237] C. Singer , H.‐C. Töpper , T. Kutsch , R. Schuster , R. Koerver , and R. Daub , “Hydrolysis of Argyrodite Sulfide‐Based Separator Sheets for Industrial All‐Solid‐State Battery Production,” ACS Applied Materials & Interfaces 14, no. 21 (2022): 24245. 35471027 10.1021/acsami.2c01099

[238] Y. Zhu and Y. Mo , “Materials Design Principles for Air‐Stable Lithium/Sodium Solid Electrolytes,” Angewandte Chemie International Edition 59, no. 40 (2020): 17472–17476, 10.1002/anie.202007621. 32597549

[239] P. Lu , L. Liu , S. Wang , et al., “Superior All‐Solid‐State Batteries Enabled by a Gas‐Phase‐Synthesized Sulfide Electrolyte with Ultrahigh Moisture Stability and Ionic Conductivity,” Advanced Materials 33, no. 32 (2021): 2100921. 10.1002/adma.202100921 34218476

[240] K. T. Kim , J. Woo , Y.‐S. Kim , et al., “Ultrathin Superhydrophobic Coatings for Air‐Stable Inorganic Solid Electrolytes: Toward Dry Room Application for All‐Solid‐State Batteries,” Advanced Energy Materials 13, no. 43 (2023): 2301600.

[241] J. Xu , Y. Li , P. Lu , et al., “Water‐Stable Sulfide Solid Electrolyte Membranes Directly Applicable in All‐Solid‐State Batteries Enabled by Superhydrophobic Li^+^‐Conducting Protection Layer,” Advanced Energy Materials 12, no. 2 (2022): 2102348.

[242] B. Jang , Y. B. Song , K. H. Baeck , et al., “Revitalizing Sulfide Solid Electrolytes for All‐Solid‐State Batteries: Dry‐Air Exposure and Microwave‐Driven Regeneration,” Advanced Energy Materials 15, no. 43 (2025): 02981.

[243] W. D. Jung , M. Jeon , S. S. Shin , et al., “Functionalized Sulfide Solid Electrolyte with Air‐Stable and Chemical‐Resistant Oxysulfide Nanolayer for All‐Solid‐State Batteries,” ACS Omega 5, no. 40 (2020): 26015–26022, 10.1021/acsomega.0c03453. 33073128 PMC7558032

[244] T. Kim , K. Kim , S. Lee , G. Song , M. S. Jung , and K. T. Lee , “Thermal Runaway Behavior of Li_6_PS_5_Cl Solid Electrolytes for LiNi_0.8_Co_0.1_Mn_0.1_O_2_ and LiFePO_4_ in All‐Solid‐State Batteries,” Chemistry of Materials 34, no. 20 (2022): 9159–9171.

[245] T. Kim , H. Chang , G. Song , et al., “Critical Factors Contributing to the Thermal Runaway of Thiophosphate Solid Electrolytes for All‐Solid‐State Batteries,” Advanced Functional Materials 34 (2024): 2404806, 10.1002/adfm.202404806.

[246] X. Rui , D. Ren , X. Liu , et al., “Distinct Thermal Runaway Mechanisms of Sulfide‐Based All‐Solid‐State Batteries,” Energy & Environmental Science 16, no. 8 (2023): 3552–3563.

[247] M. T. Hasan , A. S. J. Alujjage , B. S. Vishnugopi , et al., “Role of External Pressure in Thermal Stability of Solid‐State Batteries,” ACS Energy Letters 10, no. 11 (2025): 5762–5770.

[248] T. A. Yersak , H. J. Gonzalez Malabet , V. Yadav , N. P. W. Pieczonka , W. Collin , and M. Cai , “Flammability of Sulfide Solid‐State Electrolytes β‐Li_3_PS_4_ and Li_6_PS_5_Cl: Volatilization and Autoignition of Sulfur Vapor—New Insight into All‐Solid‐State Battery Thermal Runaway,” Journal of Energy Chemistry 102 (2025): 651–660, 10.1016/j.jechem.2024.11.031.

[249] Y. Wu , W. Zhang , X. Rui , et al., “Thermal Runaway Mechanism of Composite Cathodes for All‐Solid‐State Batteries,” Advanced Energy Materials 15 (2025): 2405183, 10.1002/aenm.202405183.

[250] J. S. Kim , S. Bong , J.‐S. Kim , et al., “Thermal Runaway in Sulfide‐Based All‐Solid‐State Batteries: Risk Landscape, Diagnostic Gaps, and Strategic Directions,” Advanced Energy Materials 15 (2025): 03593, 10.1002/aenm.202503593.

[251] J. Wang , K. Yang , S. Sun , et al., “Advances in Thermal‐Related Analysis Techniques for Solid‐State Lithium Batteries,” InfoMat 5, no. 4 (2023): 12401.

